# Climatic and socioeconomic effects on land cover changes across Europe: Does protected area designation matter?

**DOI:** 10.1371/journal.pone.0219374

**Published:** 2019-07-17

**Authors:** Niels Hellwig, Ariane Walz, Danijela Markovic

**Affiliations:** 1 Institute of Environmental Sciences and Geography, Potsdam University, Potsdam, Germany; 2 Faculty of Business Management and Social Sciences, Osnabrück University of Applied Sciences, Osnabrück, Germany; Kerala Forest Research Institute, INDIA

## Abstract

Land cover change is a dynamic phenomenon driven by synergetic biophysical and socioeconomic effects. It involves massive transitions from natural to less natural habitats and thereby threatens ecosystems and the services they provide. To retain intact ecosystems and reduce land cover change to a minimum of natural transition processes, a dense network of protected areas has been established across Europe. However, even protected areas and in particular the zones around protected areas have been shown to undergo land cover changes. The aim of our study was to compare land cover changes in protected areas, non-protected areas, and 1 km buffer zones around protected areas and analyse their relationship to climatic and socioeconomic factors across Europe between 2000 and 2012 based on earth observation data. We investigated land cover flows describing major change processes: urbanisation, afforestation, deforestation, intensification of agriculture, extensification of agriculture, and formation of water bodies. Based on boosted regression trees, we modelled correlations between land cover flows and climatic and socioeconomic factors. The results show that land cover changes were most frequent in 1 km buffer zones around protected areas (3.0% of all buffer areas affected). Overall, land cover changes within protected areas were less frequent than outside, although they still amounted to 18,800 km^2^ (1.5% of all protected areas) from 2000 to 2012. In some parts of Europe, urbanisation and intensification of agriculture still accounted for up to 25% of land cover changes within protected areas. Modelling revealed meaningful relationships between land cover changes and a combination of influencing factors. Demographic factors (accessibility to cities and population density) were most important for coarse-scale patterns of land cover changes, whereas fine-scale patterns were most related to longitude (representing the general east/west economic gradient) and latitude (representing the north/south climatic gradient).

## Introduction

Land use change is a global phenomenon and among the greatest current threats to ecosystems and the services they provide [1–4]. Even biodiversity hotspots are under intensified pressures [5] due to combined effects of anthropogenic land use practices and climate change [6–9]. Newbold et al. [10] suggested that the intactness of biodiversity already falls below the planetary boundary (i.e. the safe limit for ecosystems and humanity) for large parts of the world, which might trigger serious consequences for the functioning of the global Earth system [11–15]. Furthermore, several studies reported a positive relationship between environmental heterogeneity and biodiversity [16–20]. Altogether, land cover changes (e.g. induced by deforestation, land degradation, desertification, and urbanisation), biodiversity losses, and anthropogenic climate change are all interconnected processes that experience the impacts of profound transformation of the global environment.

In order to counteract the loss of ecosystem functions, it is required to establish effective protected areas in regions of high diversity all over the world [2,21–23]. With regard to habitat and biodiversity conservation, the effectiveness of existing protected areas is limited, since a large part of them is under intense human pressure [24]. For example, a recent investigation on forest losses in protected areas revealed that protection did not reduce forest losses in several parts of the world including Europe [25]. Additionally, legal restrictions of anthropogenic land use activities are bound to the designated protected areas, although habitats and ecosystems usually span larger areas. Thus, the effectiveness of protected areas may also be compromised by changing land use practices in the surrounding areas (hereafter referred to as the “buffers around protected areas”) [26–28]. In particular, regions that are densely populated and intensely used (such as most parts of Central Europe) are exposed to a high land use pressure. In such regions, the question arises whether land cover changes in the surroundings of protected areas are significantly higher than inside those protected areas. Due to interaction processes, this would potentially pose a threat to ecological functions provided in those protected areas.

Earth observation satellites provide valuable data for the analysis of ecosystem functions and land cover changes [29,30]. For example, Hansen et al. [31] analysed Landsat data to reveal global patterns of forest cover change during the 21st century. On a European scale, CORINE Land Cover data provide a granular reference for the analysis of land cover changes (e.g. [32,33]). Land cover data have proven to reveal natural dynamics as well as human-induced pressures, relevant also for conservation in- and outside protected areas [33,34]. However, until recently there have been only few studies that explicitly analysed land cover transitions and their relationship to climatic or socioeconomic factors with a focus on the pan-European network of protected areas [25,35]. Climatic and socioeconomic effects on land cover changes may be manifold, depending on the kind of change process (e.g. urbanisation, afforestation) [32] and on the spatial scale (e.g. local scale, regional scale) [36,37]. Therefore, it would be advantageous to analyse those effects separately for different change processes and spatial scales.

The objective of this study is to compare land cover transitions across Europe between 2000 and 2012 and to analyse the main climatic and socioeconomic factors that drive these changes using a statistical data mining approach. We focus on three area types: (1) protected areas (Natura 2000 sites and nationally designated protected areas), (2) non-protected areas, and (3) 1 km buffers around protected areas. Specifically, for the three area types, we aim to (i) compare patterns of land cover change for biogeographical regions and countries and (ii) assess climatic and socioeconomic effects on land cover changes across Europe at the local and regional scale.

## Materials and methods

### Study area

The study covers ten biogeographical regions across 39 European countries including all member states of the European Union (EU-28), Albania, Bosnia and Herzegovina, Iceland, Kosovo, Liechtenstein, Macedonia, Montenegro, Norway, Serbia, Switzerland, and Turkey (Table 1, S1 Fig). Territories located on remote islands or continents were excluded. This analysis includes 1,470 regions on Level 3 of the European Nomenclature of Territorial Units for Statistics (NUTS3, regional scale; S1 Appendix) and 127,097 Local Administrative Units (LAU, local scale), chosen due to the high availability of socioeconomic data for the whole of Europe. Countries, where spatial subdivisions were lacking (Kosovo and Serbia for NUTS3 level; Albania, Bosnia and Herzegovina, and Montenegro for NUTS3 and LAU levels), were included as individual regions.

**Table 1:** Total modelled areas (km^2^) of area types per biogeographical region [40] and country.

Biogeographical region / Country	Protected areas	Non-protected areas	1 km protected area buffers	Total modelled area
**Alpine**	235,370	413,274	74,866	648,643
**Anatolian**	677	419,867	330	420,544
**Arctic**	19,208	85,805	3,286	105,013
**Atlantic**	175,321	678,088	180,607	853,409
**Black Sea**	6,936	110,056	2,010	116,993
**Boreal**	91,057	803,686	174,362	894,743
**Continental**	382,600	1011,812	290,638	1394,412
**Mediterranean**	265,537	922,890	130,800	1188,427
**Pannonian**	27,957	120,362	30,025	148,319
**Steppic**	8,043	29,048	5,743	37,091
**Albania**	4,832	23,959	1,727	28,791
**Austria**	23,649	60,299	15,266	83,948
**Belgium**	7,178	23,486	12,595	30,664
**Bosnia and Herzegovina**	120	51,092	811	51,212
**Bulgaria**	38,475	72,513	20,528	110,989
**Croatia**	21,472	35,127	9,696	56,600
**Cyprus**	3,485	5,764	968	9,249
**Czech Republic**	17,303	61,567	14,981	78,870
**Denmark**	7,161	36,014	35,814	43,175
**Estonia**	8,760	36,575	18,074	45,335
**Finland**	50,354	287,264	51,120	337,617
**France**	139,700	409,383	83,483	549,082
**Germany**	134,681	223,056	123,784	357,737
**Greece**	46,036	85,701	18,480	131,736
**Hungary**	20,799	72,215	21,320	93,013
**Iceland**	17,899	84,803	3,224	102,701
**Ireland**	9,775	60,181	17,029	69,957
**Italy**	64,547	236,072	46,528	300,620
**Kosovo**	1,253	9,603	553	10,856
**Latvia**	11,855	52,741	6,524	64,596
**Liechtenstein**	69	91	77	160
**Lithuania**	11,136	53,766	11,058	64,901
**Luxembourg**	1,100	1,496	992	2,595
**Macedonia**	2,042	23,394	1,171	25,436
**Malta**	95	222	193	316
**Montenegro**	6	13,873	179	13,879
**Netherlands**	6,098	31,276	5,771	37,374
**Norway**	55,356	267,666	25,058	323,024
**Poland**	123,842	188,101	46,554	311,942
**Portugal**	19,603	69,244	6,698	88,847
**Romania**	55,921	182,443	31,487	238,364
**Serbia**	5,393	72,159	4,385	77,552
**Slovakia**	18,411	30,616	9,081	49,028
**Slovenia**	10,957	9,316	5,608	20,274
**Spain**	138,794	359,736	76,412	498,530
**Sweden**	63,720	385,844	96,806	449,564
**Switzerland**	3,058	38,229	15,515	41,287
**Turkey**	1,833	778,458	1,101	780,291
**United Kingdom**	69,494	175,125	54,292	244,620
**Total**	1,216,275	4,608,494	894,950	5,824,769

For every biogeographical region, country, NUTS3 region and LAU region, we studied land cover changes within three area types: 1) protected areas, 2) non-protected areas, and 3) 1 km buffers around protected areas. Protected areas included the total network of Natura 2000 sites and nationally designated areas (as obtained from [38,39]). Previous studies have shown that protected areas often span only part of those ecosystems relevant for the provided ecological functions [26,28]. Hamilton et al. [29] developed land use scenarios focusing on buffer zones of 5 km, 25 km, and 75 km around protected areas of the United States of America. In this study, in view of the dense European network of protected areas, we decided to analyse separately land cover changes in 1 km buffer zones around protected areas.

### Land cover data

The data basis for land cover analyses consisted of the CORINE Land Cover Change layers in a 100 m grid resolution for the periods 2000–2006 and 2006–2012 [41]. These data sets were used to calculate land cover flows as aggregated groups of land cover changes from 2000 to 2012. Feranec et al. [32] discussed the quality of CORINE land cover change layers and concluded that they were suitable for the calculation of land cover flows. The overall thematic accuracy of CORINE land cover change data (2000) was found to be high [42].

Based on Feranec et al. [32], the following land cover flows (LCFs) were considered: urbanisation (LCF1), intensification of agriculture (LCF2), extensification of agriculture (LCF3), afforestation (LCF4), deforestation (LCF5), formation of water bodies (LCF6). All land cover flows refer to the analysis of transitions between CORINE land cover classes as introduced by Feranec et al. [32]. For example, intensification of agriculture (LCF2) involves land cover changes from natural and semi-natural areas to agricultural lands and from less intense agricultural uses to more intense ones (i.e. from pastures to heterogeneous agricultural areas to arable land to permanent crops). On the contrary, extensification of agriculture (LCF3) involves land cover changes from more intense agricultural uses to less intense ones. Land cover flows (LCF1-LCF6) were calculated as areas per area type (i.e. protected areas, non-protected areas, 1 km protected area buffers) for every biogeographical region, country, NUTS3 region, and LAU region. The maps presented in S1 Appendix show the percentages of LCFs per NUTS3 region from 2000 to 2012.

### Climatic and socioeconomic data

We analysed multiple factors to evaluate the relationship between land cover changes from 2000 to 2012 and the spatiotemporal climatic and socioeconomic dynamics. Climatic factors included changes of temperature, precipitation, and frequency of wet days during the 20th century. Socioeconomic factors included accessibility to cities (i.e. the travel time to cities), population density, and changes of gross development product and population from 2000 to 2012. Furthermore, we considered longitude and latitude as proxies of the general climatic patterns and the protected area proportion as a proxy of the human response to the habitat and biodiversity loss. All considered factors and the corresponding data sources are listed in Table 2. The spatial distributions of the factors as aggregated per NUTS3 regions are illustrated in S2 Appendix.

**Table 2:** Climatic and socioeconomic factors included in models of land cover flows.

Factor	Symbol	Source
Change of maximum temperature of the warmest month (1951–2000 vs. 1900–1950)	Tmax_Ch	[43]
Change of minimum temperature of the coldest month (1951–2000 vs. 1900–1950)	Tmin_Ch	[43]
Change of annual precipitation sum (1951–2000 vs. 1900–1950)	Precip_Ch	[43]
Change of frequency of wet days (1951–2000 vs. 1900–1950)	Wetfreq_Ch	[43]
Accessibility to cities (travel distance in 2015)	Access	[44]
GDP change (2000–2012, only at NUTS3 level)	GDP_Ch	[45]
Population change (2000–2012)	Pop_Ch	[46,47]
Population density 2011/2012	PopDens	[46,47]
Longitude	Long	-
Latitude	Lat	-
Protected area proportion 2018	PA_Prop	[38,39]

### Statistical modelling

For statistical modelling, the land cover flows were coded binary, i.e. whenever any land cover flow LCFx exceeded a certain percent threshold within any NUTS3 or LAU region, the value 1 was assigned for LCFx to this region, and otherwise the value 0. In order to account for the different value distributions of land cover flows (S3 Appendix), the following values of the LCF distributions were examined as thresholds: 0, 0.05 quantile, 0.1 quantile, and 0.25 quantile.

Models in this study included (as illustrated in S2 Fig): a) three area types (protected areas, non-protected areas, 1 km protected area buffers), b) two spatial aggregation levels (NUTS3, LAU), c) six land cover flows (LCF1-LCF6), and d) four thresholds for binary coding of the LCF distributions (0, 0.05 quantile, 0.1 quantile, 0.25 quantile).

**Fig 1:**
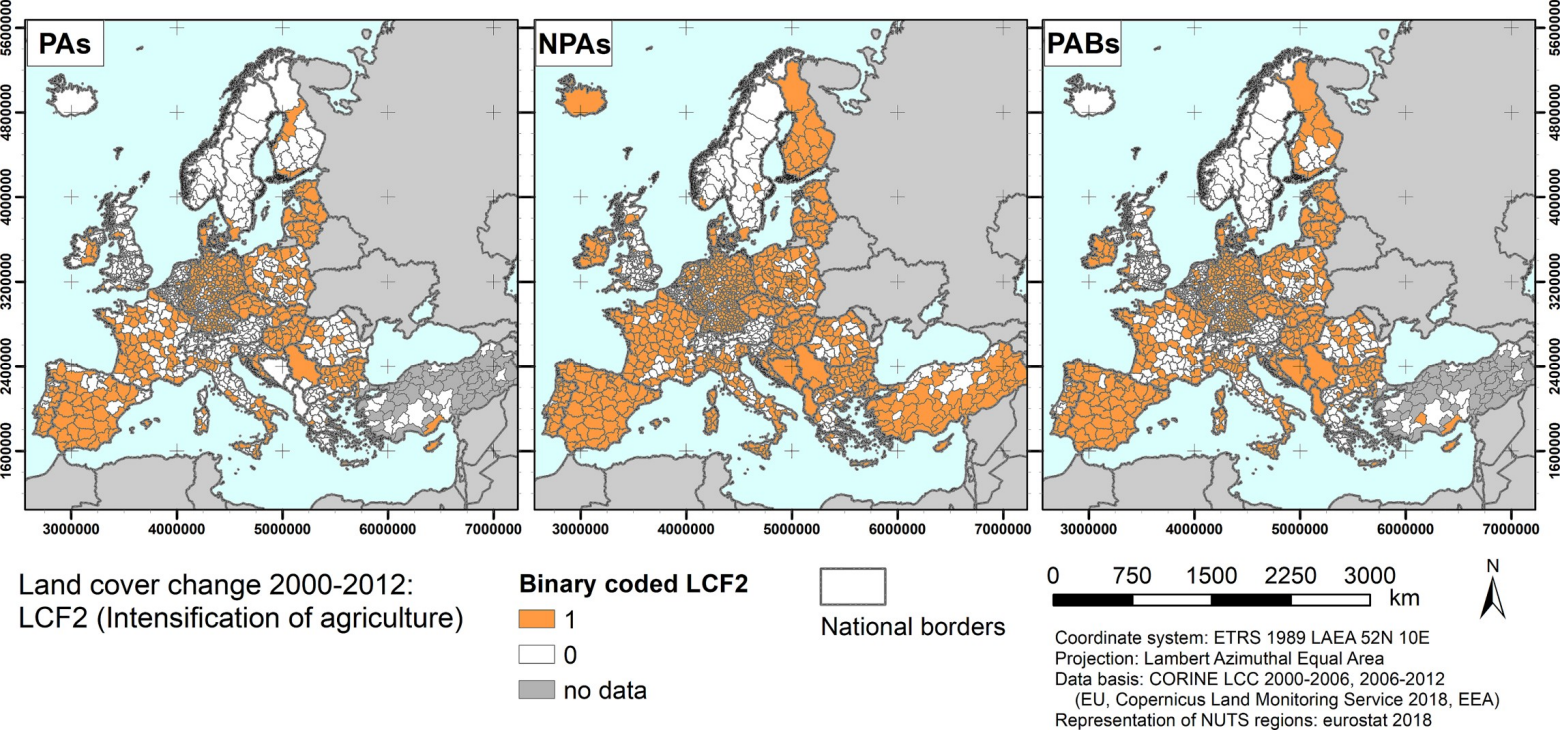
Binary coded LCF2 (intensification of agriculture) patterns for the three area types (PAs = protected areas, NPAs = non-protected areas, PABs = 1 km protected area buffers) on NUTS3 level using the 0.1 quantiles of the LCF2 distribution.

As an example, Fig 1 shows the patterns of LCF2 (intensification of agriculture) for the three area types on NUTS3 level using the 0.1 quantiles. The tables in S3 Appendix list the numbers of zeros and ones that characterised the LCF distributions for modelling.

Statistical models of land cover changes were implemented using boosted regression trees. Boosted regression trees are an ensemble modelling technique implying a set of binary decision trees, which are built sequentially based on a boosting algorithm. This algorithm modifies the training data for tree building according to the residuals of the previous trees [48,49]. Overall, boosted regression trees provide a powerful framework for modelling both non-linear relationships and interactions between variables in large environmental datasets. For that reason, boosted regression trees have been widely used for ecological modelling purposes during the last years (e.g. [50–54]). As compared to other modelling methods, boosted regression trees deal relatively well with collinearity among the predictors [55]. In this study, the predictors show low to moderate correlations (S4 Appendix).

All models were implemented with the statistical software R (version 3.5.0) [56] and the R package gbm for boosted regression trees [57]. Models were selected by optimising the model parameters number of trees, tree complexity, learning rate, and bag fraction with 10-fold cross-validation. The total number of models was 2880, comprising 20 repetitions of 144 models (3 area types × 2 spatial aggregation levels × 6 land cover flows × 4 thresholds = 144).

Modelling at NUTS3 level included all 1,470 regions; at LAU level, a random sample of 20,000 regions was used. The model performance was evaluated estimating the area under the receiver operating characteristic (ROC) curve using the R package ROCR [58]. The relative importance of any climatic and socioeconomic factor (i.e. the sum of improvements in squared error induced by all tree splits of the factor in the boosted regression trees) was calculated according to Friedman [59] using the R package gbm (method summary.gbm) [57].

## Results

### Geographic patterns of land cover change

**Fig 2:**
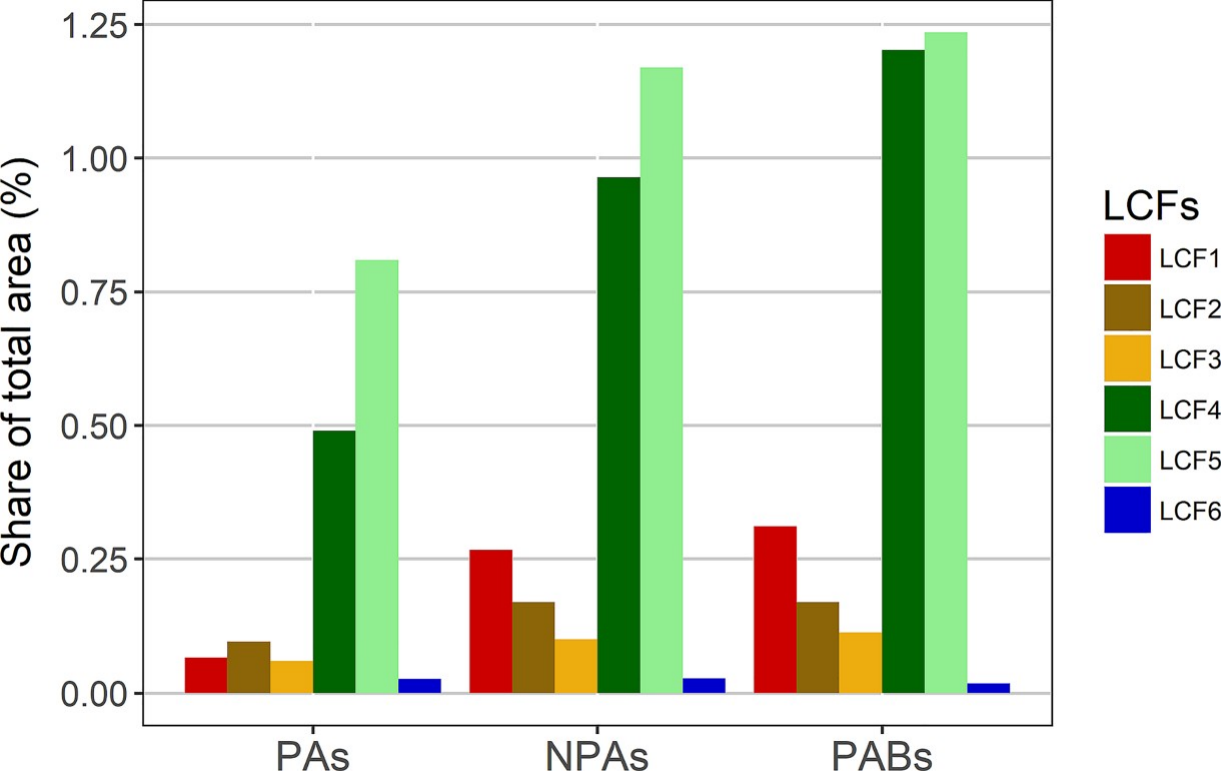
Land cover flows (LCFs) in protected areas (PAs), non-protected areas (NPAs), and 1 km protected area buffers (PABs) as shares of total modelled areas. LCF1 = Urbanisation, LCF2 = Intensification of agriculture, LCF3 = Extensification of agriculture, LCF4 = Afforestation, LCF5 = Deforestation, LCF6 = Formation of water bodies.

The total areas affected by land cover flows from 2000 to 2012 amounted to 143,044 km^2^ (ca. 2.5% of the study area). These changes occurred on 18,778 km^2^ in protected areas (ca. 1.5% of all modelled protected areas) and on 124,266 km^2^ in non-protected areas (ca. 2.7% of all modelled non-protected areas). In 1 km protected area buffers, areas of 27,275 km^2^ were affected by land cover flows (ca. 3.0% of all 1 km protected area buffers) (Table A in S5 Appendix). Afforestation and deforestation (LCF4, LCF5) accounted for the largest part of land cover flows in all area types (Fig 2, see also S5 Appendix). Urbanisation (LCF1) took the third place ahead of intensification of agriculture (LCF2) and extensification of agriculture (LCF3) in non-protected areas, whereas these three land cover flows almost went head to head in protected areas.

**Fig 3:**
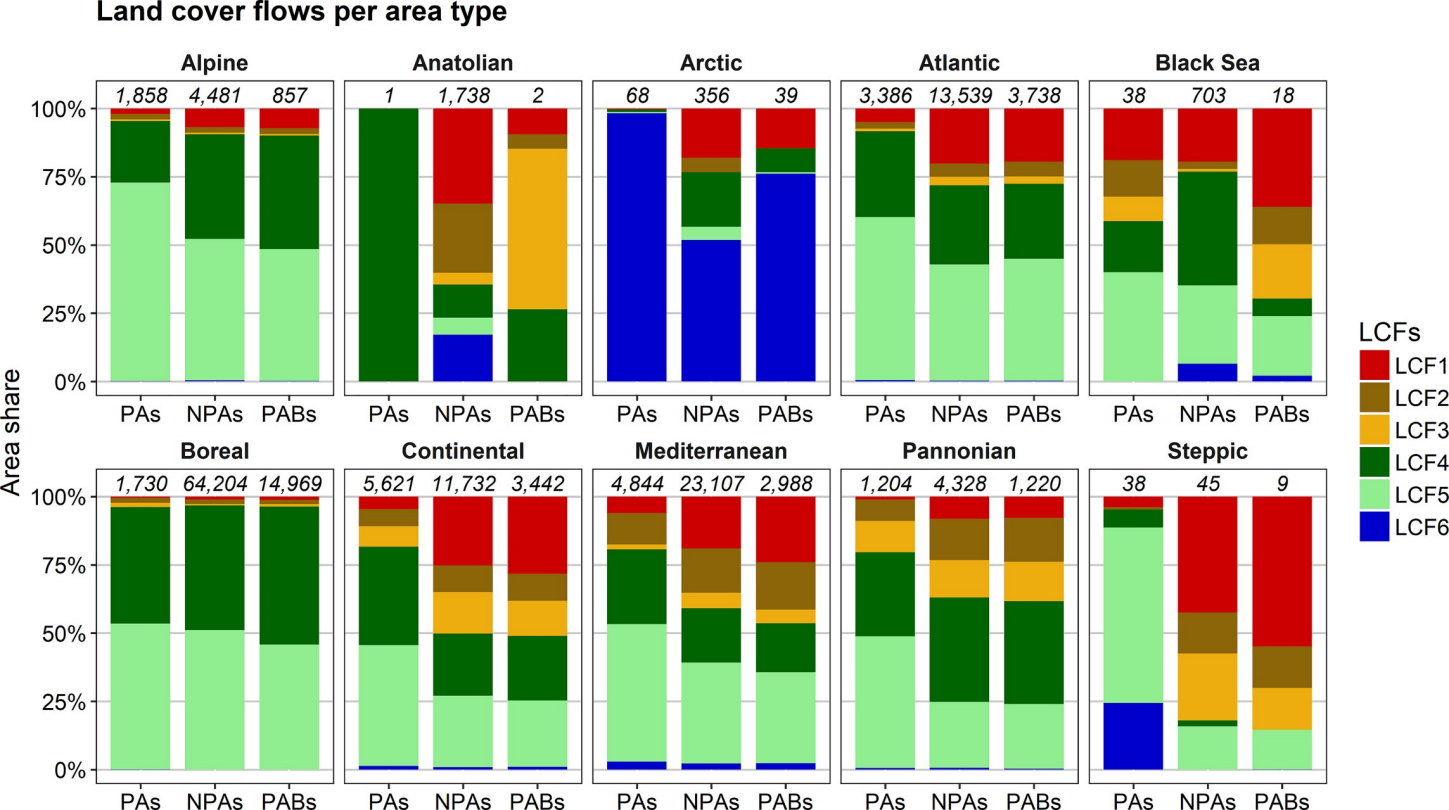
Patterns of land cover flows (LCFs) in protected areas (PAs), non-protected areas (NPAs), and 1 km protected area buffers (PABs) depending on the biogeographical region. Total areas of all land cover flows per area type and biogeographical region (rounded to km^2^) are given in italics above the bars. LCF1 = Urbanisation, LCF2 = Intensification of agriculture, LCF3 = Extensification of agriculture, LCF4 = Afforestation, LCF5 = Deforestation, LCF6 = Formation of water bodies.

Patterns of land cover flows varied significantly between different biogeographical zones (Fig 3). For most biogeographical regions, the major part of land cover changes between 2000 and 2012 involved afforestation (LCF4) and deforestation (LCF5), where total forest cover declined in most cases (LCF4 < LCF5). Intensification and extensification of agriculture (LCF2, LCF3) and particularly urbanisation (LCF1) were more frequent processes relative to all land cover changes out of protected areas than within protected areas. Except for the Alpine, the Boreal, and the Pannonian zones, urbanisation accounted for at least 20 to 25% of all land cover flows in non-protected areas. On the contrary, excluding the Black Sea region, urbanisation accounted for maximum 6% of all land cover flows within protected areas.

At the country level, patterns of land cover flows from 2000 to 2012 showed a high diversity (S3 Fig, see also S5 Appendix). In general, urbanisation (LCF1) and intensification of agriculture (LCF2) tended to be the dominant processes of land cover changes in Central European countries such as Germany, the Netherlands, and Slovenia (more than 60% of all land cover flows in non-protected areas). Within protected areas, urbanisation and intensification of agriculture were less frequent. However, these processes still accounted for approximately 25% of all land cover flows within protected areas in Albania, Croatia, Cyprus, Germany, and Spain. In most of the other countries, afforestation (LCF4) and deforestation (LCF5) were the most common processes. There were countries with a distinct loss of forest cover (LCF4 < LCF5, e.g. Austria, Latvia, Norway, Romania, United Kingdom) and with a distinct gain of forest cover (e.g. Sweden).

### Model results

**Fig 4:**
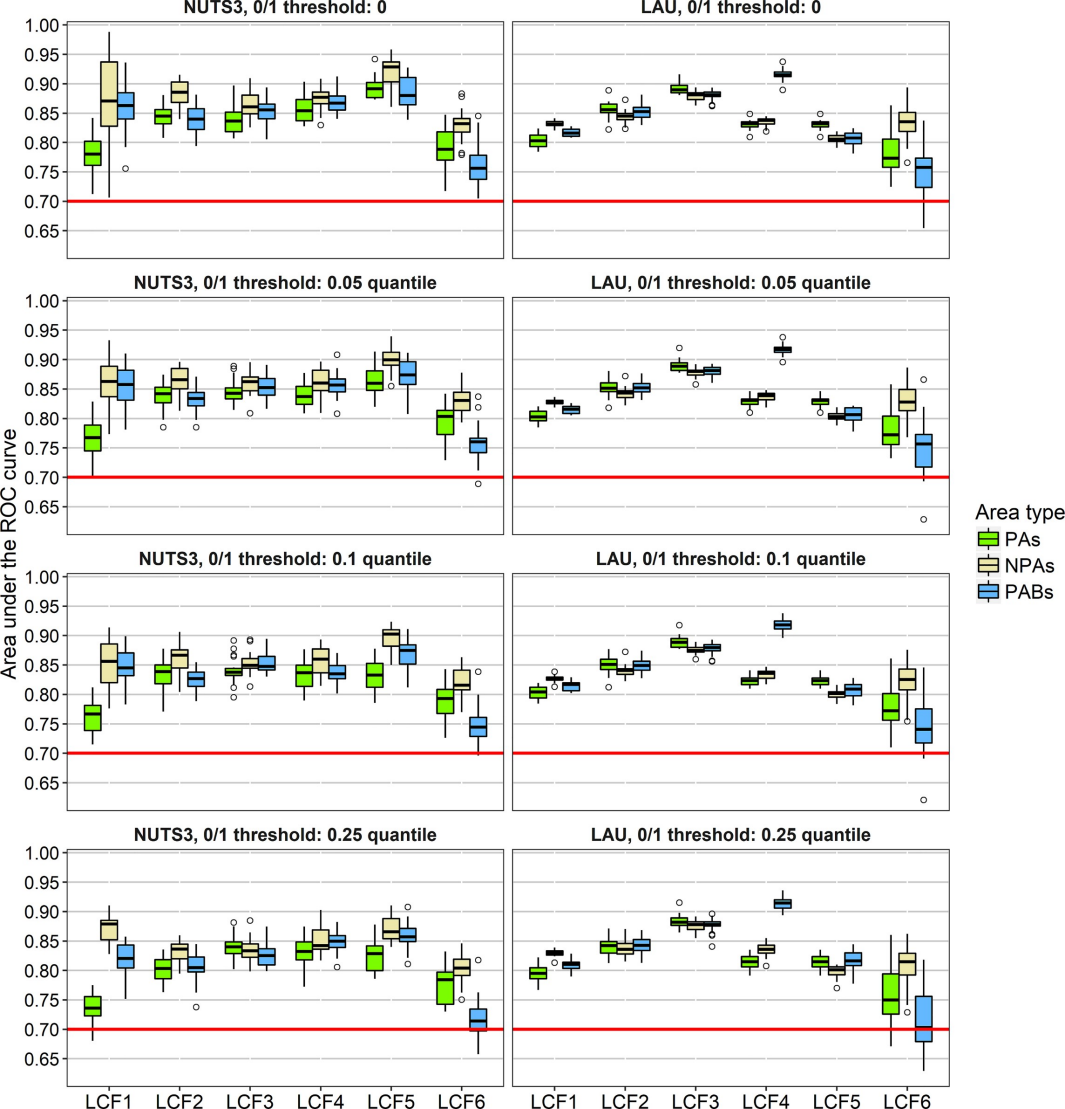
Performances of boosted regression trees at NUTS3 and LAU level. The red line represents a threshold of 0.7 for the area under the ROC curve, which is exceeded in most model runs. LCF1 = Urbanisation, LCF2 = Intensification of agriculture, LCF3 = Extensification of agriculture, LCF4 = Afforestation, LCF5 = Deforestation, LCF6 = Formation of water bodies.

Fig 4 illustrates the performances of boosted regression trees distinguished by land cover flows, area types, thresholds for binary coding, and scale (NUTS3 vs. LAU) as evaluated by the area under the receiver operating characteristic (ROC) curve (AUC). All models showed median AUC values above 0.7. Except for LCF6 (formation of water bodies) and LCF1 (urbanisation) inside protected areas (especially at the NUTS3 level), median AUC values even exceeded 0.8.

In general, patterns of AUC values varied only slightly between the different thresholds for binary coding. However, there were distinct differences between the analysed scales (NUTS3 and LAU). At NUTS3 level unlike at LAU level, AUC values tended to be highest for non-protected areas–in some cases the differences between non-protected areas and the other area types were significant. Furthermore, the model performances were distinctly more stable for all land cover flows other than LCF6 at LAU level than at NUTS3 level (as indicated by the tiny boxes in Fig 4).

**Fig 5:**
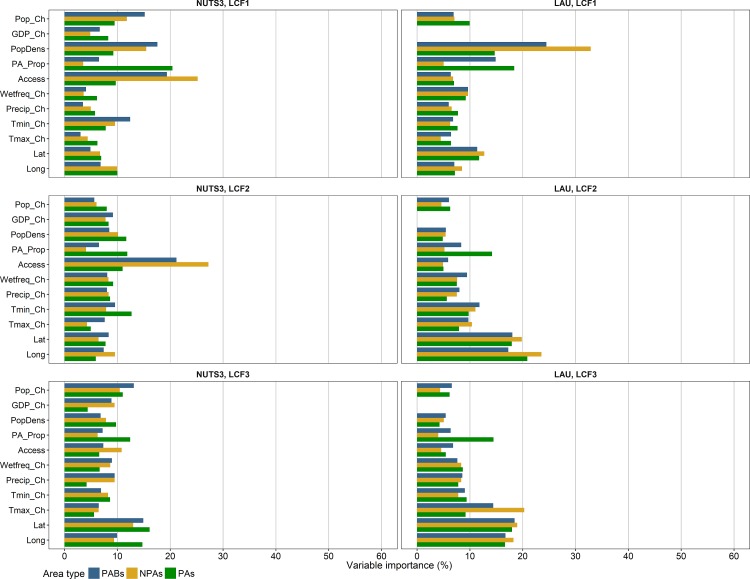
Relative importances of climatic and socioeconomic factors (see Table 2) in boosted regression trees for LCF1-LCF3 (0.1 quantile) at NUTS3 and LAU level. LCF1 = Urbanisation, LCF2 = Intensification of agriculture, LCF3 = Extensification of agriculture.

**Fig 6:**
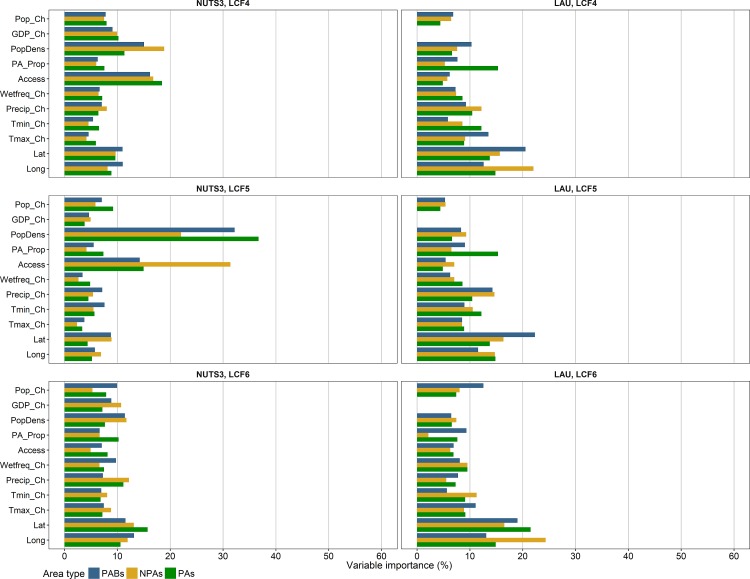
Relative importances of climatic and socioeconomic factors (see Table 2) in boosted regression trees for LCF4-LCF6 (0.1 quantile) at NUTS3 and LAU level. LCF4 = Afforestation, LCF5 = Deforestation, LCF6 = Formation of water bodies.

Figs 5 and 6 show the relative importances of climatic and socioeconomic factors in the boosted regression trees with 0.1 quantiles as thresholds for binary coding. In general, all factors seemed to be relevant for the model results, as all factors were closer to the average (~10% importance) than to 0% in the majority of models. However, the relative factor importances varied between the area types, the NUTS3 and LAU level, and especially the land cover flows. The variation in the distribution of factor importances between the models with different thresholds for binary coding was relatively small (S4 Fig). This especially applied to the LAU level, which corresponded to a higher stability of model results as compared to the NUTS3 level.

At NUTS3 level, the most important factors were accessibility to cities and population density with values from ~15–20% up to 60% in the following models: urbanisation (LCF1) in non-protected areas and in protected area buffers, intensification of agriculture (LCF2) in non-protected areas and in protected area buffers (only accessibility to cities), afforestation (LCF4), and deforestation (LCF5). Moreover, for LCF1 in protected areas, the protected area proportion had a relative importance of ~20%. At LAU level, the protected area proportion was among the most important factors for all models of LCF1-LCF5 in protected areas (~15–20%). Besides, the factors with relatively high importances were population density for LCF1 (~15–30%), longitude and latitude for LCF2-LCF6 (~15–20%), and the change of maximum temperature for LCF3 in non-protected areas and in protected area buffers (~15–20%).

## Discussion

### Land cover changes inside and around protected areas

**Fig 7:**
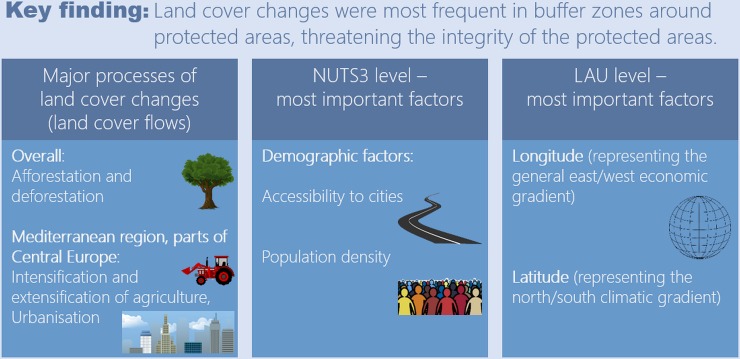
Key findings on land cover changes across Europe from 2000 to 2012 and the most important associated climatic and socioeconomic factors.

Overall, land cover changes for land cover flows LCF1-LCF6 were ca. 143,000 km^2^ (approximately equivalent to the sum of the areas of Hungary and Slovakia) between 2000 and 2012 (Table A in S5 Appendix). In protected areas, human-induced land cover change (e.g. forestry, cultivation of land, livestock production, urban development) is rare. Accordingly, protected areas were less affected by land cover changes than non-protected areas in Europe from 2000 to 2012. In particular, inside protected areas, land cover changes related to urbanisation (LCF1) were only about a quarter of the frequency of those outside (Fig 2). Thus, the designation of protected areas was effective in reducing, though not completely preventing, anthropogenic land cover changes. Protected areas underwent approximately 18,800 km^2^ of land cover changes in total (LCF1-LCF6), where afforestation and deforestation accounted for the largest parts (Fig 7, Table A in S5 Appendix). This is in line with the results from Heino et al. [25], who found significant forest losses in protected areas throughout Europe from 2000 to 2012. In fact, relative forest losses in protected areas partly exceeded those in non-protected areas at national scale [25]. However, previous studies generally indicate a varying effectiveness of protected areas in preventing land cover changes. For example, several habitat losses attributed to (human-induced) land cover change have been recorded in protected areas in South Asia during the last century [60]. In East Africa, only National Parks were shown to be successful in forest conservation, whereas other protected areas lost forest areas between 2001 and 2009 [61]. In a study of land cover changes in Spain, Martínez-Fernández et al. [34] found considerably higher changes in Natura 2000 areas as compared to nationally designated protected areas. Lu et al. [62] analysed land cover change in protected areas across the United States of America. Although those areas experienced little land cover change after they had been established, a clear effect of the establishment of protected areas could not be found [62].

Among all investigated area types, the rate of land cover changes was highest in 1 km protected area buffers (Fig 7), although relatively similar to the rate of land cover changes in all non-protected areas (Fig 2). This is in line with Davis & Hansen [63] who found that land use changes around National Parks of the United States of America exceeded national rates of land use change. Moreover, the simulation study by Dorning et al. [64] indicated that the establishment of protected areas leads to increasing urban sprawl outside of these areas. Ecological processes in protected areas and in surrounding areas are interrelated, since species’ habitats and ecosystem units often span larger areas than those designated for protection [26,28]. Thus, land use activities in buffer areas around protected areas may disturb ecological functions and processes inside protected areas, for example due to landscape fragmentation and pollution. Populations of animals with wide home ranges face the highest risk of extinction due to disturbances in those buffer areas [65].

Based on the results of our study, future conservation management should consider expanding the network of protected areas to protect ecosystems from degradation caused by anthropogenic land cover change. Moreover, highly affected protected areas and buffer zones need to be prioritised in the monitoring process, eventually tightening legal measures to prevent land use activities in these areas. Although our results point to the threats that land cover change poses to protected areas throughout Europe, the consequences of land cover change for protection goals need to be evaluated at the level of each individual protected area. Specific land cover changes in and around protected areas might be acceptable or even desirable, especially those associated with natural dynamics or with ecosystem recovery due to law enforcement.

### Modelling land cover changes

The analysis of land cover changes was based on land cover flows as aggregates of transitions between CORINE land cover classes from 2000 to 2012. Errors induced by misclassification of forests and agricultural areas (especially at the levels of subcategorisation of CORINE classes) or too low resolution of CORINE land cover change layers have been pointed out [32,42]. However, those errors are unlikely to affect our results substantially, due to the continental extent of our study and the binary coding of land cover flows per spatial unit (NUTS3 and LAU regions).

The aggregation of land cover changes to land cover flows enables one to differentiate frequent processes triggering land cover change (e.g. urbanisation, intensification of agriculture). Nevertheless, information gets partly lost, as there may be overlaps between processes. For example, transitions from forest to urban areas are attributed to LCF1 (urbanisation) but not to LCF5 (deforestation). Thus, a comparison of LCF4 (afforestation) and LCF5 (deforestation) values does not inform about total forest gains or losses. However, the classification of land cover flows according to Feranec et al. [32] proved to be useful, as land cover changes are attributed to their (main) drivers (in case of a change from forest to urban areas the driver is rather the construction of new urban facilities than the removal of forest). Additionally, land cover flows are designed to integrate gross land cover changes, thus this approach avoids the tendency to underestimate land cover changes when regarding net changes of land cover classes [66]. LCF1 (urbanisation) and LCF2 (intensification of agriculture) are obviously related to anthropogenic drivers. On the contrary, drivers of LCF3 (extensification of agriculture), LCF4 (afforestation), and LCF5 (deforestation) may be anthropogenic as well as non-anthropogenic. For example, these flows may be caused by changing land management practices, natural succession, logging, natural disasters, pests, or climate change. Water bodies (LCF6) may originate either from construction or as proglacial lakes.

The study of land cover flows not only enhances the understanding of land cover changes and underlying mechanisms, but also allows to gain new insights into the relationship between land cover dynamics and ecosystem functions. For example, studying land cover flows enabled Muñoz‐Rojas et al. [67] to draw inferences on the changes of soil organic carbon stocks. Other recent studies on continental or global scale have analysed changes of specific land cover/land use classes (e.g. [31,68–70]) or land cover changes classified as archetypical change trajectories [71].

Model results at the LAU level showed a higher stability as compared to the NUTS3 level (Fig 4), corresponding with a much higher number of spatial units (20,000 out of 127,097 LAU regions versus 1,470 NUTS3 regions). Different influencing factors proved important at both scales. This implies that all factors with high relative importance values at either of both scales were related to the respective land cover flows, but they are effective at different spatial scales. For most models, accessibility to cities and population density were more important factors at NUTS3 level than at LAU level (Figs 5 and 6). Land cover changes at LAU level, on the contrary, were more related to the general east/west economic gradient (longitude) and north/south climatic gradient (latitude) than at NUTS3 level. These findings indicate that a mixture of climatic and socioeconomic factors influenced patterns of land cover changes differently at both model scales (Fig 7). To gain a comprehensive understanding of the effects of spatial scale on the analysis of factors important for land cover change, future studies need to address cross-scale interactions between drivers and change patterns, taking into account the implications of grouping spatial data differently.

### Climatic and socioeconomic effects on land cover

Patterns of land cover change varied according to biogeographic regions. Afforestation (LCF4) and deforestation (LCF5) were the dominant land cover flows in most biogeographic regions. Urbanisation (LCF1) was more pronounced in the Atlantic and Continental regions as compared to the Alpine and Boreal regions, where afforestation and deforestation had the highest dominance of all regions (Fig 3). These findings correspond to the patterns of land cover change in Europe between 1950 and 2000 [72]. In most parts of Europe, afforestation and deforestation are primarily due to forestry, as the patterns of LCF4 and LCF5 coincide (Figures D and E in S1 Appendix) and these patterns are similar to those of wood production [68,73]. However, large-scale changes of forest cover are also triggered by fires, especially in the Mediterranean region, where droughts increase the risk of summer fires [74]. Urbanisation (LCF1) proceeds as urban concentration or urban sprawl depending on local and regional development planning. For example, Ahrens & Lyons [75] found a higher rate of urban sprawl, i.e. higher distances between new artificial areas and already existing artificial areas, for Ireland as compared to other parts of Europe. Patterns of intensification and extensification of agriculture (LCF2, LCF3) are diverse across Europe (Fig 3) confirming the findings of previous studies that showed significant variation of agricultural development at regional levels [76,77]. Especially in mountain areas, agricultural land has been abandoned in many places [69,78,79]. In the traditional agricultural landscapes of Slovakia, financial unprofitability and cultural changes were reported as main drivers for land abandonment, especially in areas with steep slopes and unfertile soils [80]. The formation of water bodies (LCF6) was dominant in the Arctic region (Fig 3). This reflects the increased formation of proglacial lakes due to glacier melt in this region [81,82].

In this study, we found that land cover changes across Europe were related to climatic and socioeconomic factors between 2000 and 2012. This is in line with recent studies focusing on these relationships on various regional to global scales (e.g. [25,35,69,83–86]). As species’ habitats are disturbed by land cover changes, these correlations imply that also patterns of species distributions are affected by climatic and socioeconomic factors. This is in line with Mouchet et al. [87] who found that species richness of terrestrial vertebrates was related to climatic and land use variables. Climate change affects ecosystem functions not only directly by changing temperature and precipitation regimes, but also indirectly by increasing the risk of land cover change. However, the factor importances in our models demonstrate that land cover changes were more related to the location within Europe (latitude and longitude) than to the climatic changes during the 21st century (especially at LAU level). Moreover, land cover changes in our models were distinctly related to population density and accessibility to cities (especially at NUTS3 level: LCF1, LCF2, LCF4, and LCF5) (Fig 5). Luck [88] reported that a high population density is closely connected with changes of the biodiversity status. Thus, especially those protected areas that are located close to areas suitable for intense land use are exposed to a high risk of species extinction [88].

Our model results show evidence for the relationships of climatic and socioeconomic factors to land cover changes between 2000 and 2012, but they do not reveal causalities, i.e. why land covers change or why land uses are transformed in a specific case. On local scales, main drivers of land cover changes are very specific, even though these are connected with regional- to global-scale climatic and socioeconomic factors [89].

## Conclusions

The main objective of this study was to analyse land cover transitions and related climatic and socioeconomic factors across Europe between 2000 and 2012. Among all investigated area types, land cover changes were most frequent in 1 km protected areas buffers (3.0% of all buffer areas affected). Due to strong interactions between protected areas and their surroundings, these findings indicate a threat also to ecological functions provided inside protected areas. The designation of protected areas was effective in reducing, though not completely preventing, anthropogenic land cover changes; 1.5% of all protected areas were still affected by land cover change.

Overall, afforestation and deforestation were found to be the most prominent land cover changes. However, in the Mediterranean region and in parts of Central Europe, urbanisation as well as intensification and extensification of agriculture were major processes of land cover change. The percentages of purely anthropogenic processes (urbanisation and intensification of agriculture) were generally lower inside protected areas than outside. Nevertheless, these processes accounted for ca. 25% of all land cover flows within protected areas in Albania, Croatia, Cyprus, Germany, and Spain.

Variations of land cover changes were generally related to a combination of climatic and socioeconomic factors. Demographic factors (accessibility to cities and population density) were most important for patterns of land cover change at NUTS3 level, whereas patterns at LAU level were most related to longitude (representing the general east/west economic gradient) and latitude (representing the north/south climatic gradient).

In total, the analysis has shown that land cover change across Europe between 2000 and 2012 was associated with the absolute degree of climatic and socioeconomic influences, rather than with the corresponding temporal changes of climatic and socioeconomic factors. As climate change and socioeconomic development may involve higher and spatially correlated extremes in the future, our findings suggest a high potential impact on land cover change, and thus high vulnerability of ecosystem services. Outside of protected areas, land cover change is a ubiquitous phenomenon, which massively alters ecological interactions. Therefore, to ensure protected area integrity, the edge effects of land cover changes in protected area buffer zones on the protected ecosystems need to be regularly monitored and assessed.

## Supporting information

S1 Appendix Maps of land cover flows (LCF1-LCF6) from 2000 to 2012 per NUTS3 region. (PDF)

S2 Appendix Maps of the spatial distribution of climatic and socioeconomic factors per NUTS3 region. (PDF)

S3 Appendix Distribution of input values for modelling of land cover flows (LCF1-LCF6) with different thresholds for binary coding. (PDF)

S4 Appendix Correlations of model covariates. (PDF)

S5 Appendix Spatial distribution of land cover flows from 2000 to 2012. (PDF)

S1 Fig Biogeographical regions and countries of the study area. (TIF)

S2 Fig Overview of models implemented in this study (PAs = protected areas, NPAs = non-protected areas, PABs = 1 km protected area buffers). (TIF)

S3 Fig Patterns of land cover flows (LCFs) in protected areas (PAs), non-protected areas (NPAs), and 1 km protected area buffers (PABs) depending on the country. Area share values refer to the total areas of all land cover flows per area type and country. Total area values (in km^2^) are given in S5 Appendix.
(TIF)

S4 Fig Relative importances of climatic and socioeconomic factors (see Table 2) in boosted regression trees for LCF1-LCF3 at NUTS3 and LAU level. (TIF)
