# Supplementary material for: Climatic and socioeconomic effects on land cover changes across Europe: Does protected area designation matter?
Source: PLoS One. 2019 Jul 17;14(7):e0219374. doi: 10.1371/journal.pone.0219374 (PMC6636817; doi:10.1371/journal.pone.0219374)
Supplement: S1 Appendix — (PDF) [file pone.0219374.s001.pdf]

S1 Appendix: Maps of land cover flows (LCF1-LCF6) from 2000 to 2012 per NUTS3 region.

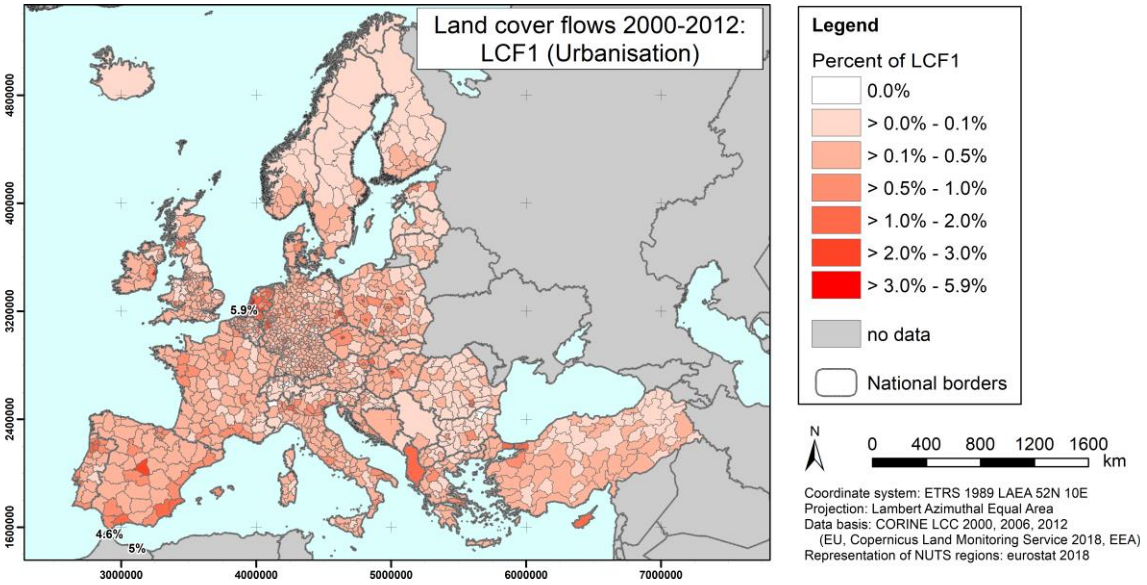

Fig A. Share of LCF1 (Urbanisation) 2000 to 2012 per NUTS3 region.

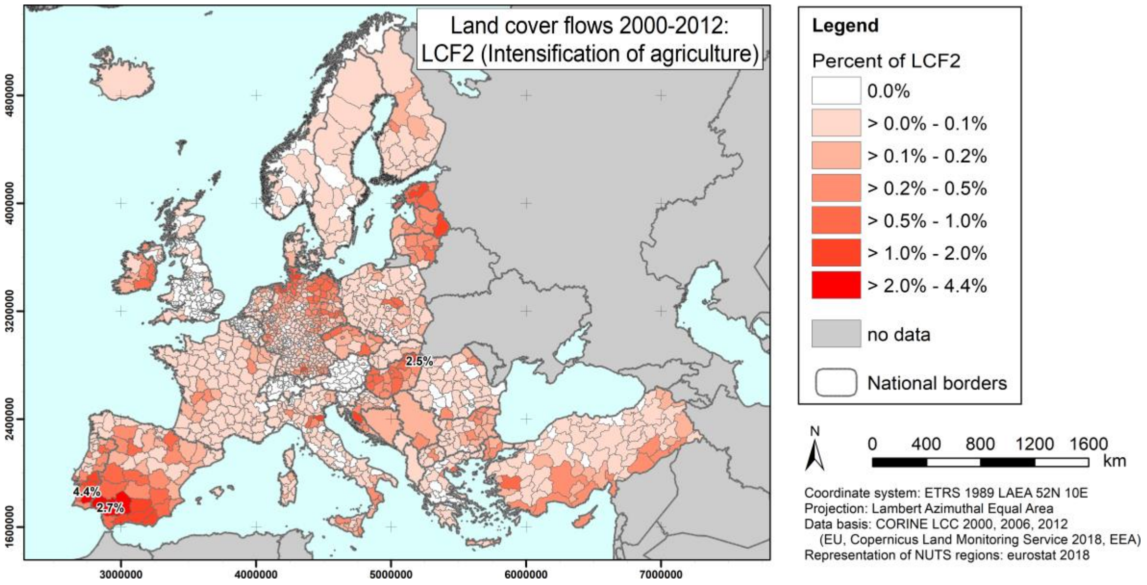

Fig B. Share of LCF2 (Intensification of agriculture) 2000 to 2012 per NUTS3 region.

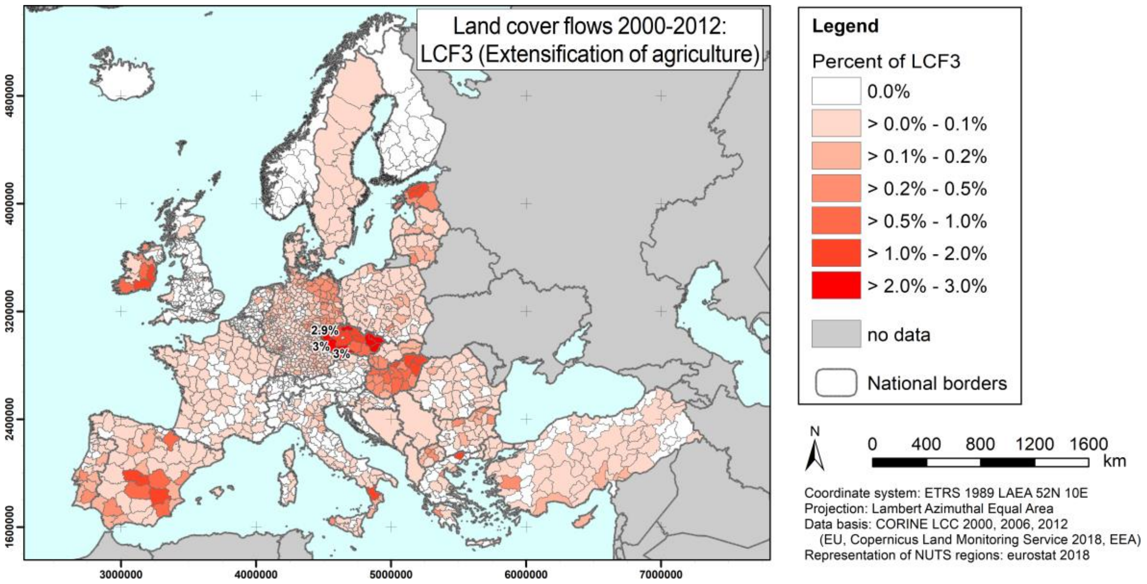

Fig C. Share of LCF3 (Extensification of agriculture) 2000 to 2012 per NUTS3 region.

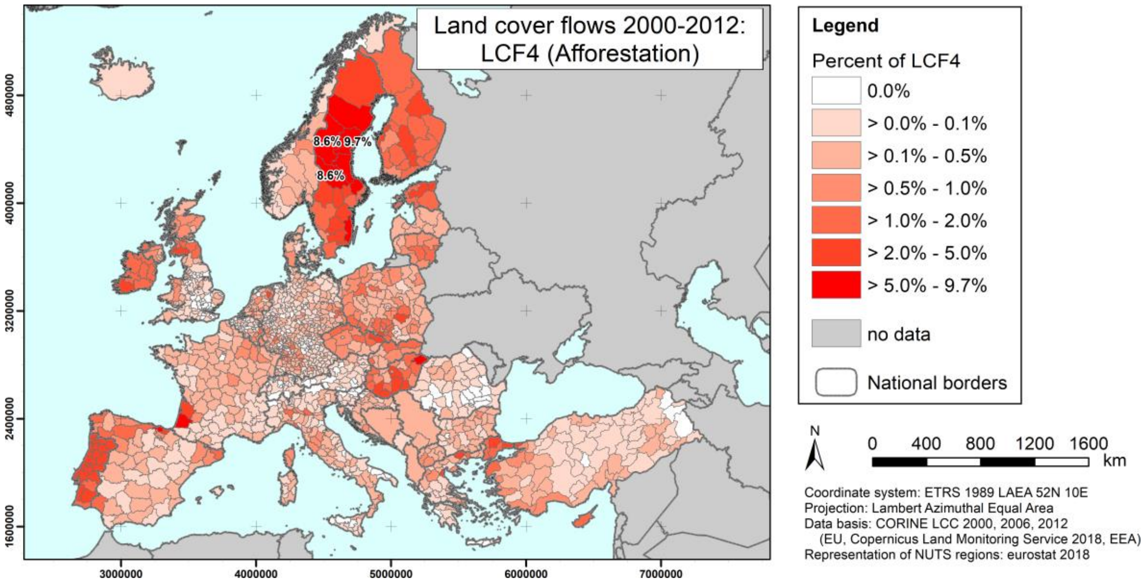

Fig D. Share of LCF4 (Afforestation) 2000 to 2012 per NUTS3 region.

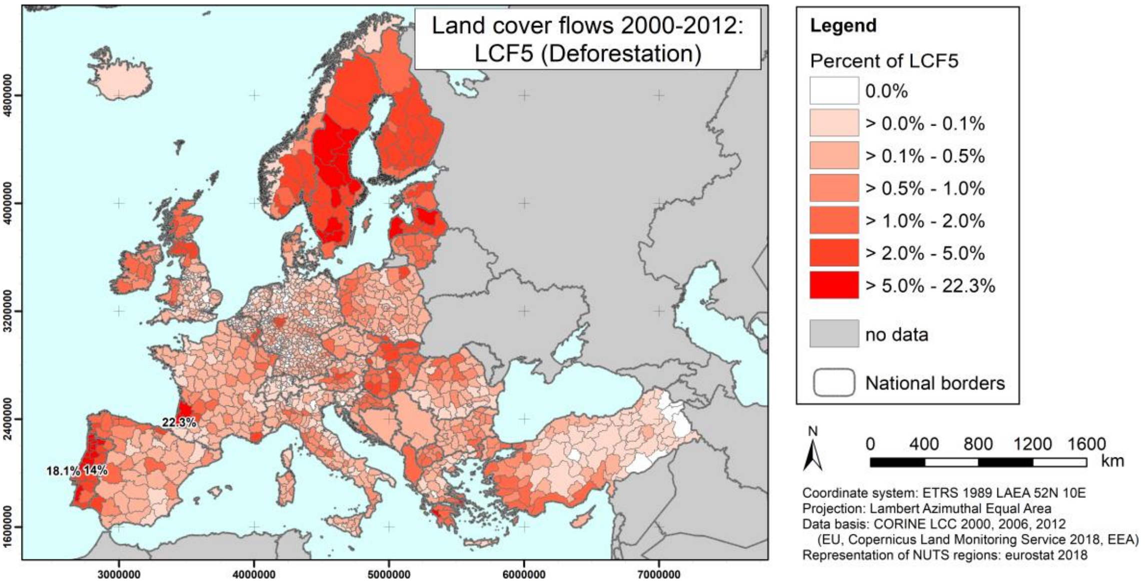

Fig E. Share of LCF5 (Deforestation) 2000 to 2012 per NUTS3 region.

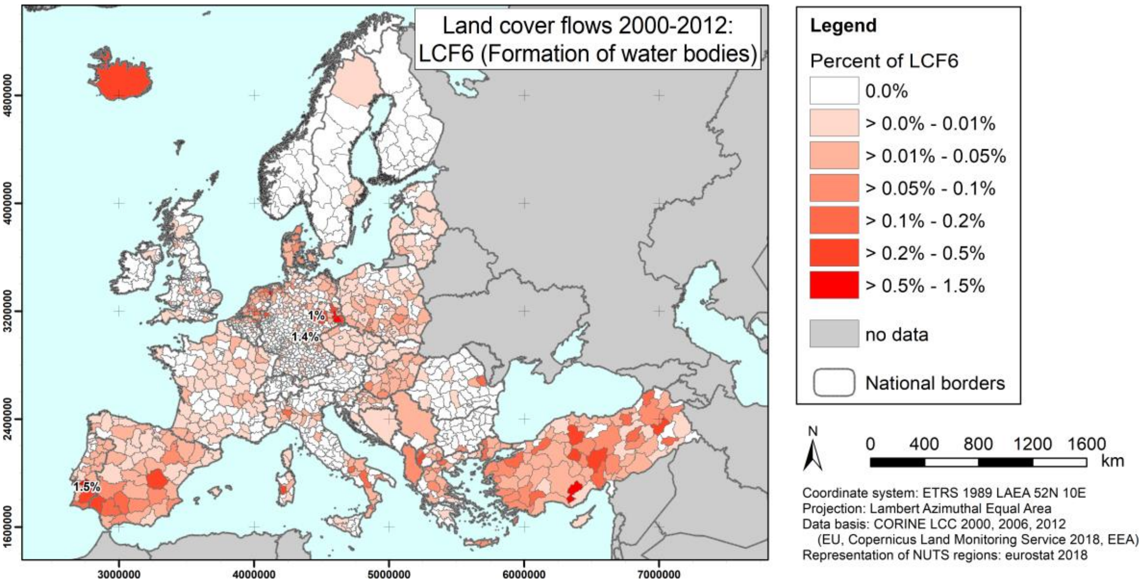

Fig F. Share of LCF6 (Formation of water bodies) 2000 to 2012 per NUTS3 region.
