# Supplementary material for: Climatic and socioeconomic effects on land cover changes across Europe: Does protected area designation matter?
Source: PLoS One. 2019 Jul 17;14(7):e0219374. doi: 10.1371/journal.pone.0219374 (PMC6636817; doi:10.1371/journal.pone.0219374)
Supplement: S2 Appendix — (PDF) [file pone.0219374.s002.pdf]

S2 Appendix: Maps of the spatial distribution of climatic and socioeconomic factors per NUTS3 region.

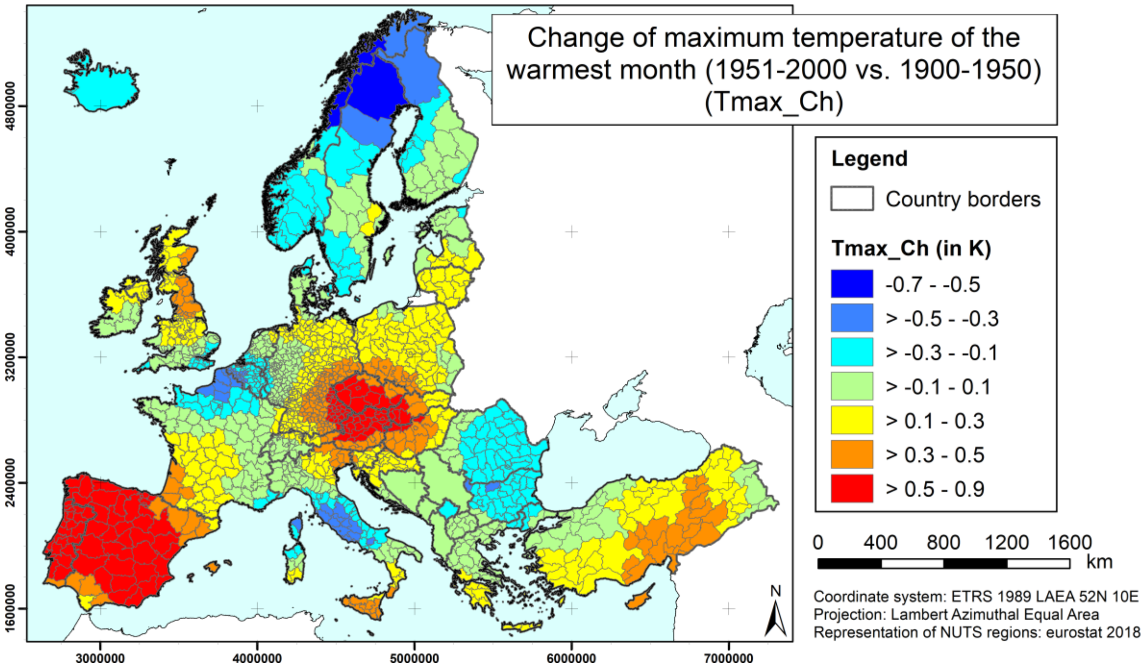

Fig A. Spatial distribution of the change of maximum temperature of the warmest month (1951-2000 vs. 1900-1950) (Tmax_Ch). Calculated based on data from Reference [42].

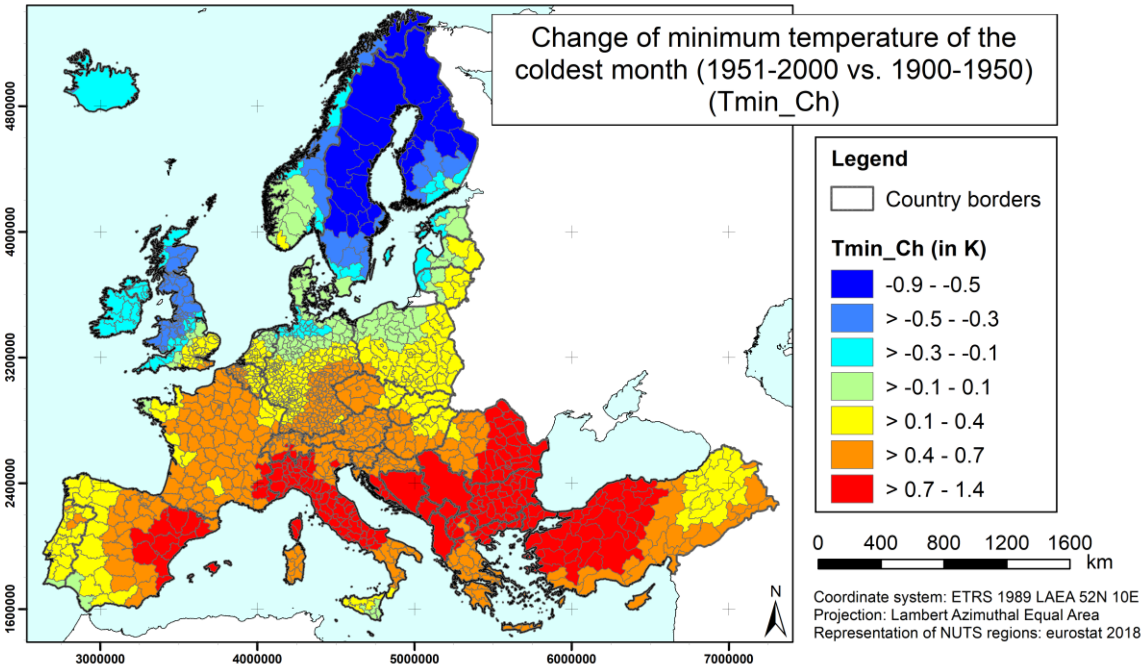

Fig B. Spatial distribution of the change of minimum temperature of the coldest month (1951-2000 vs. 1900-1950) (Tmin_Ch). Calculated based on data from Reference [42].

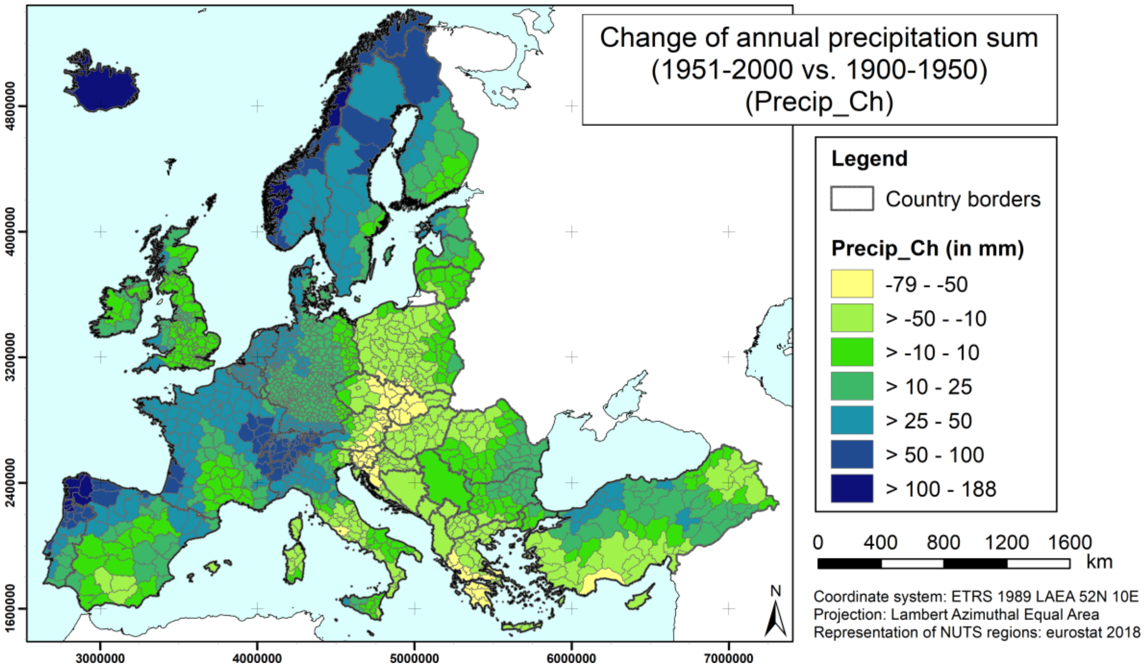

Fig C. Spatial distribution of the change of annual precipitation sum (1951-2000 vs. 1900-1950) (Precip_Ch). Calculated based on data from Reference [42]**.**

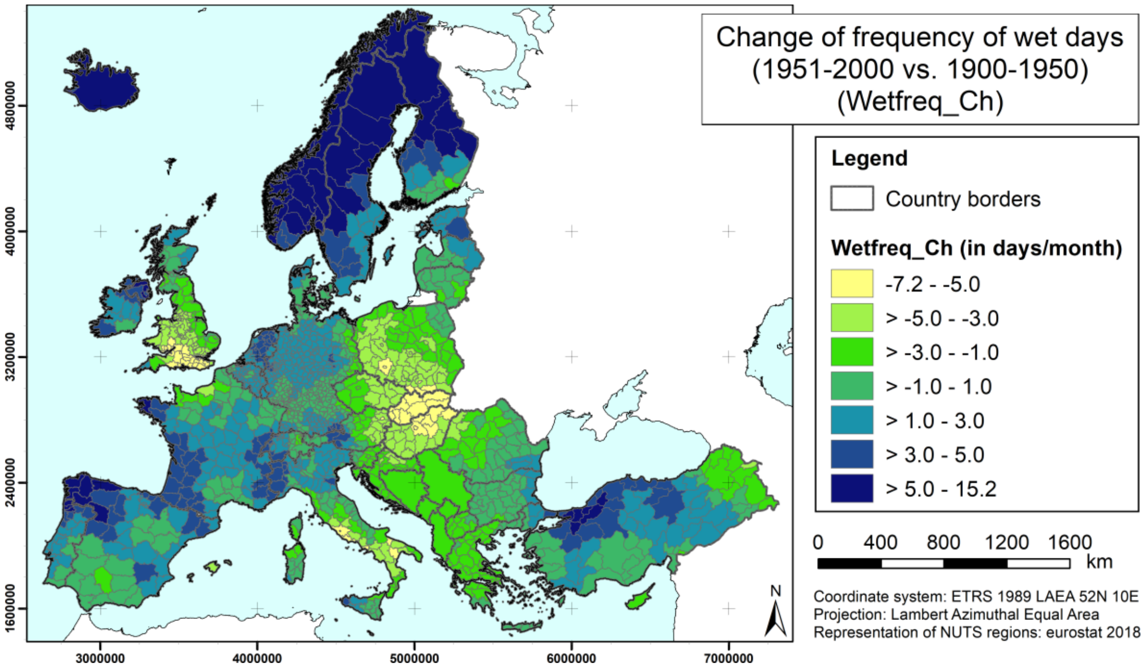

Fig D. Spatial distribution of the change of frequency of wet days (1951-2000 vs. 1900-1950) (Wetfreq_Ch). Calculated based on data from Reference [42]**.**

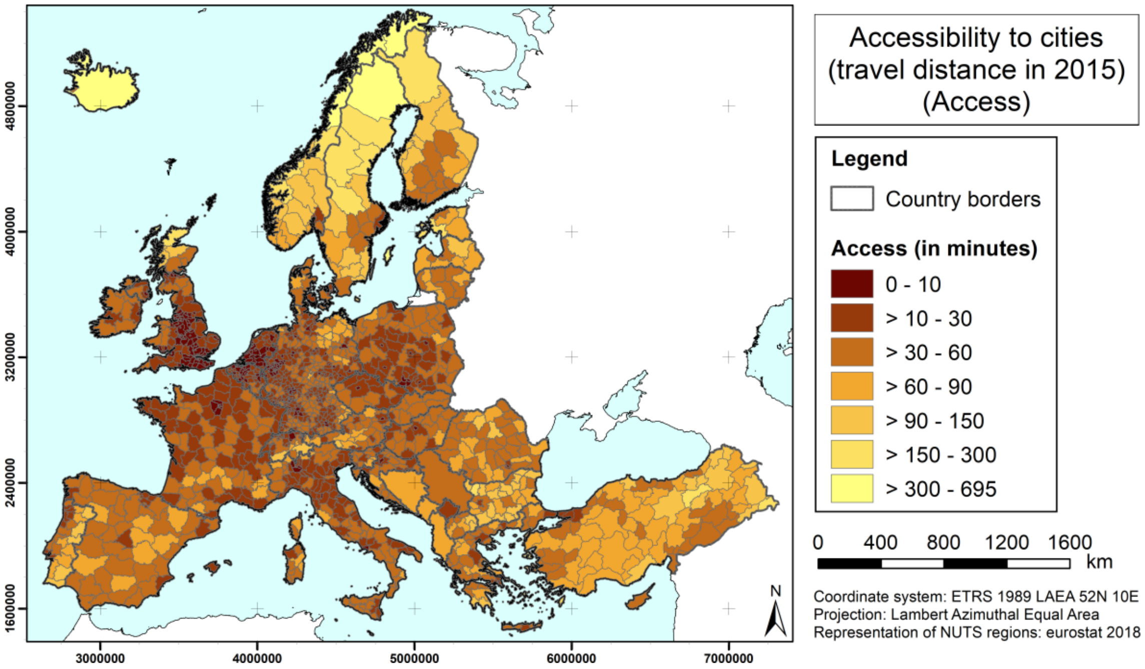

Fig E. Spatial distribution of the accessibility to cities (travel distance in 2015) (Access). Data from Reference [44]**.**

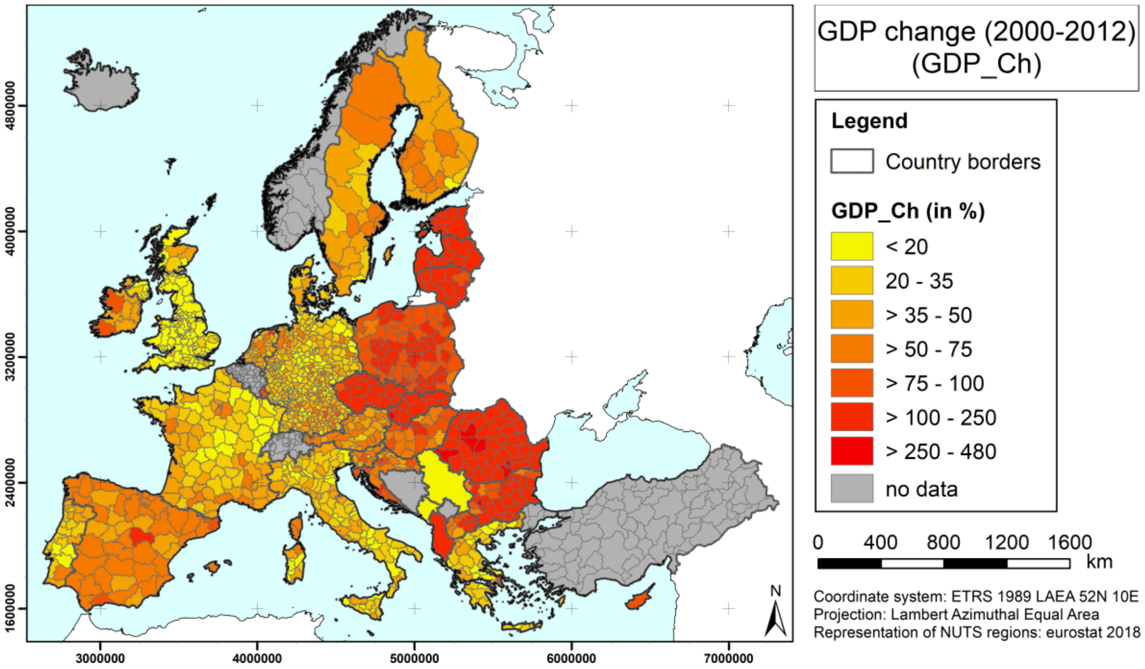

Fig F. Spatial distribution of the GDP change (2000-2012) (GDP_Ch). Calculated based on data from Reference [45].

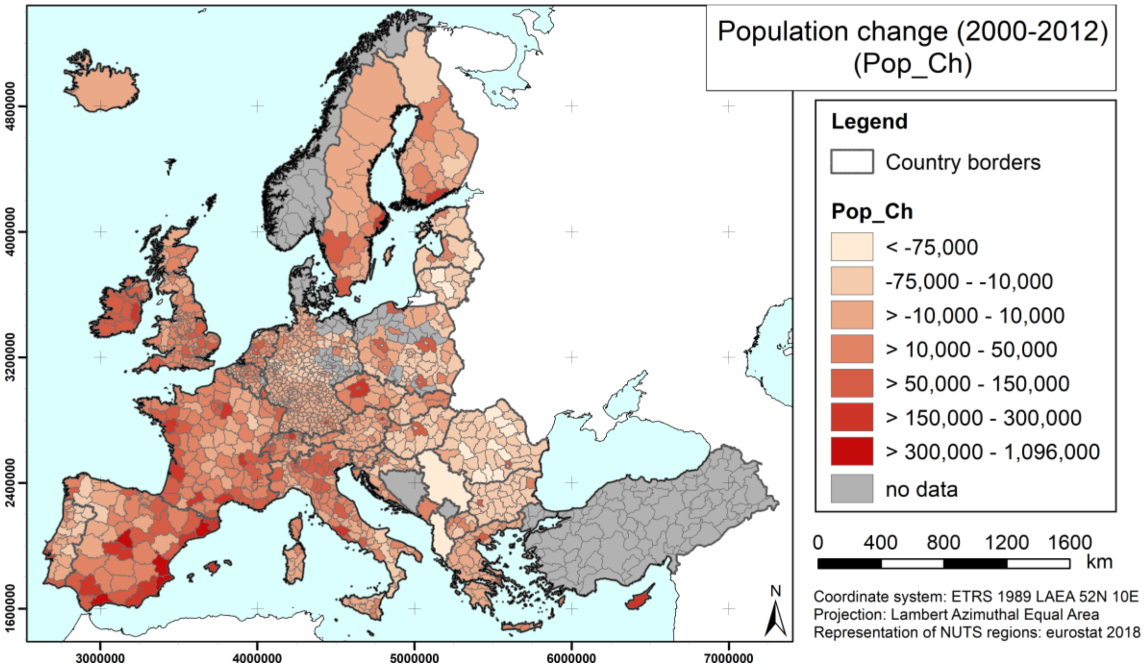

Fig G. Spatial distribution of the population change (2000-2012) (Pop_Ch). Calculated based on data from References [46,47].

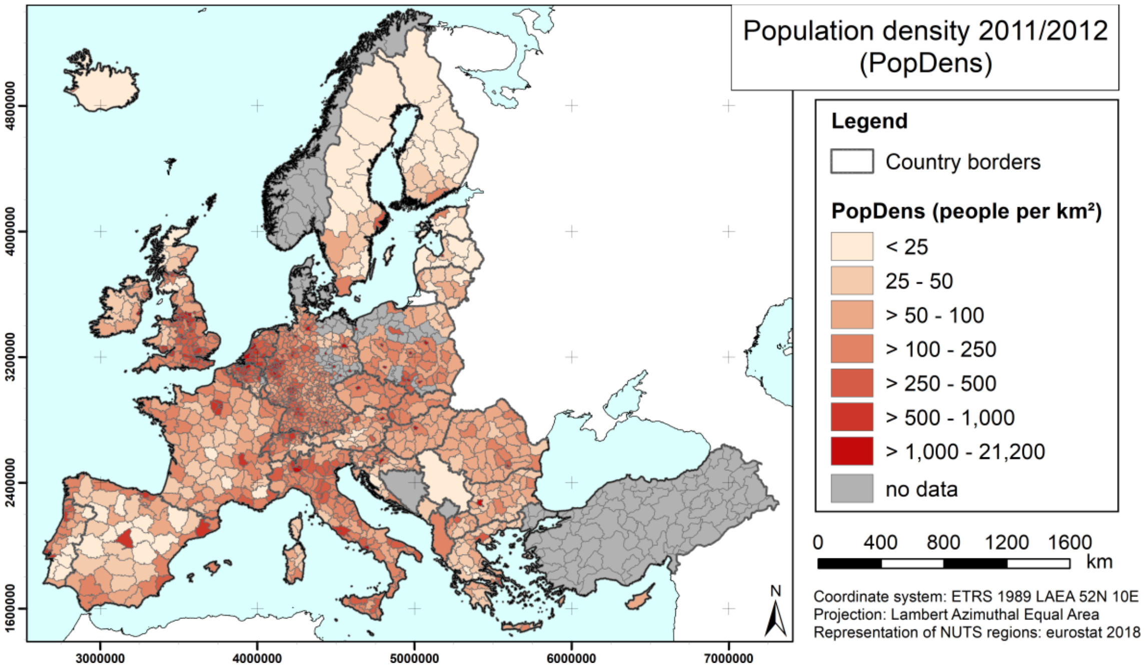

Fig H. Spatial distribution of the population density 2011/2012 (PopDens). Data from References [46,47].

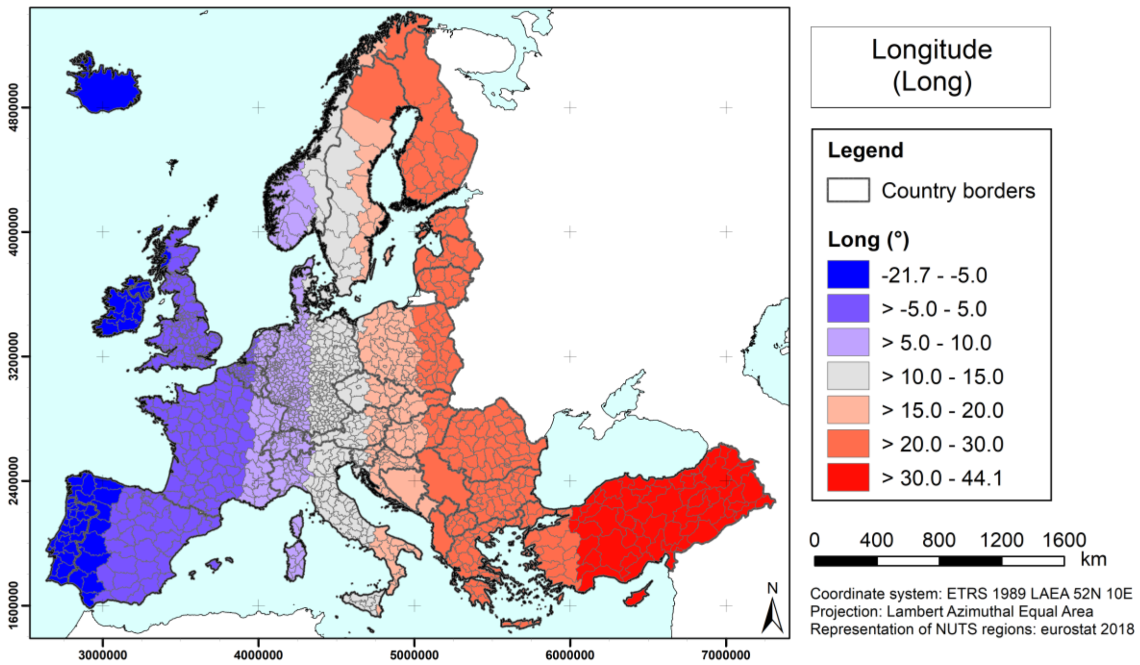

Fig I. Spatial distribution of the longitude (Long).

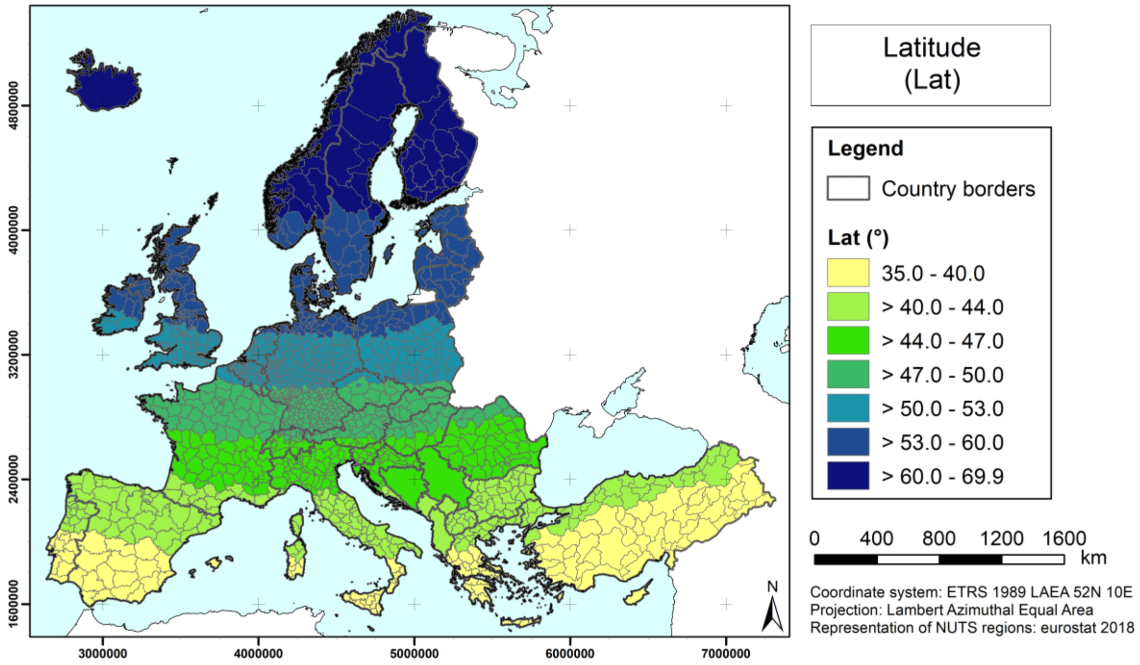

Fig J. Spatial distribution of the latitude (Lat).

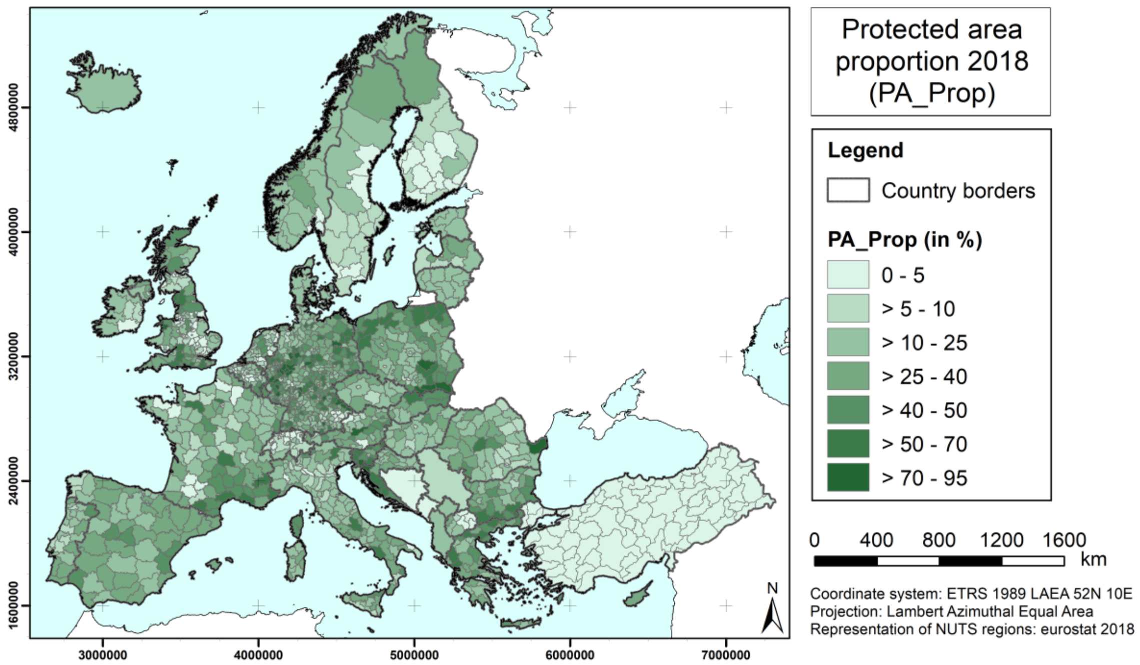

Fig K. Spatial distribution of the protected area proportion (PA_Prop). Calculated based on data from References [39,40].
