# Supplementary material for: Climatic and socioeconomic effects on land cover changes across Europe: Does protected area designation matter?
Source: PLoS One. 2019 Jul 17;14(7):e0219374. doi: 10.1371/journal.pone.0219374 (PMC6636817; doi:10.1371/journal.pone.0219374)
Supplement: S3 Appendix — (PDF) [file pone.0219374.s003.pdf]

S3 Appendix: Distribution of input values for modelling of land cover flows (LCF1-LCF6) with different thresholds for binary coding

Table A. Distribution of input values for modelling of land cover flows (LCF1-LCF6) with different thresholds for binary coding on NUTS3 level (1470 regions in total)

Land cover flow/ Threshold for binary coding	Protected areas			Non-protected areas			1 km protected area buffers		
	Zeros	Ones	No data	Zeros	Ones	No data	Zeros	Ones	No data
**LCF1**									
0	428	978	64	55	1415	0	125	1284	61
0.05 quantile	476	930	64	125	1345	0	189	1220	61
0.1 quantile	525	881	64	196	1274	0	253	1156	61
0.25 quantile	672	734	64	409	1061	0	446	963	61
**LCF2**									
0	786	620	64	512	958	0	688	721	61
0.05 quantile	817	589	64	559	911	0	724	685	61
0.1 quantile	848	558	64	607	863	0	760	649	61
0.25 quantile	941	465	64	751	719	0	868	541	61
**LCF3**									
0	956	450	64	660	810	0	829	580	61
0.05 quantile	978	428	64	700	770	0	858	551	61
0.1 quantile	1001	405	64	741	729	0	887	522	61
0.25 quantile	1068	338	64	862	608	0	974	435	61
**LCF4**									
0	457	949	64	394	1076	0	510	899	61
0.05 quantile	504	902	64	447	1023	0	555	854	61
0.1 quantile	552	854	64	501	969	0	600	809	61
0.25 quantile	694	712	64	663	807	0	735	674	61
**LCF5**									
0	375	1031	64	326	1144	0	424	985	61
0.05 quantile	426	980	64	383	1087	0	473	936	61
0.1 quantile	478	928	64	440	1030	0	522	887	61
0.25 quantile	633	773	64	612	858	0	670	739	61
**LCF6**									
0	1102	304	64	998	472	0	1154	255	61
0.05 quantile	1117	289	64	1021	449	0	1166	243	61
0.1 quantile	1132	274	64	1045	425	0	1179	230	61
0.25 quantile	1178	228	64	1116	354	0	1218	191	61

Table B. Distribution of input values for modelling of land cover flows (LCF1-LCF6) with different thresholds for binary coding on LAU level (127102 regions in total)

Land cover flow/ Threshold for binary coding	Protected areas			Non-protected areas			1 km protected area buffers		
	Zeros	Ones	No data	Zeros	Ones	No data	Zeros	Ones	No data
**LCF1**									
0	71989	4946	50167	91922	28539	6641	71391	12676	43035
0.05 quantile	72236	4699	50167	93349	27112	6641	72025	12042	43035
0.1 quantile	72483	4452	50167	94776	25685	6641	72658	11409	43035
0.25 quantile	73226	3709	50167	99057	21404	6641	74560	9507	43035
**LCF2**									
0	73346	3589	50167	108110	12351	6641	78645	5422	43035
0.05 quantile	73525	3410	50167	108727	11734	6641	78916	5151	43035
0.1 quantile	73705	3230	50167	109345	11116	6641	79187	4880	43035
0.25 quantile	74243	2692	50167	111198	9263	6641	80000	4067	43035
**LCF3**									
0	74546	2389	50167	111815	8646	6641	80324	3743	43035
0.05 quantile	74665	2270	50167	112247	8214	6641	80511	3556	43035
0.1 quantile	74785	2150	50167	112679	7782	6641	80698	3369	43035
0.25 quantile	75143	1792	50167	113976	6485	6641	81260	2807	43035
**LCF4**									
0	67959	8976	50167	101697	18764	6641	75416	8651	43035
0.05 quantile	68407	8528	50167	102635	17826	6641	75848	8219	43035
0.1 quantile	68856	8079	50167	103573	16888	6641	76284	7783	43035
0.25 quantile	70203	6732	50167	106388	14073	6641	77579	6488	43035
**LCF5**									
0	63905	13030	50167	95808	24653	6641	72697	11370	43035
0.05 quantile	64556	12379	50167	97040	23421	6641	73265	10802	43035
0.1 quantile	65208	11727	50167	98273	22188	6641	73834	10233	43035
0.25 quantile	67162	9773	50167	101973	18488	6641	75539	8528	43035
**LCF6**									
0	76264	671	50167	118926	1535	6641	83571	496	43035
0.05 quantile	76297	638	50167	119002	1459	6641	83595	472	43035
0.1 quantile	76331	604	50167	119079	1382	6641	83620	447	43035
0.25 quantile	76432	503	50167	119310	1151	6641	83695	372	43035
