# Supplementary material for: Climatic and socioeconomic effects on land cover changes across Europe: Does protected area designation matter?
Source: PLoS One. 2019 Jul 17;14(7):e0219374. doi: 10.1371/journal.pone.0219374 (PMC6636817; doi:10.1371/journal.pone.0219374)
Supplement: S4 Appendix — (PDF) [file pone.0219374.s004.pdf]

S4 Appendix: Correlations of model covariates

Table A. Correlation matrix (Pearson correlation coefficient r) of model covariates on NUTS3 level (*n* = 1470)

	Long	Lat	PA_Prop	Tmax_Ch	Tmin_Ch	Precip_Ch	Wetfreq_Ch	Access	PopDens	GDP_Ch	Pop_Ch
**Long**	1	-0.1000	0.1125	-0.2086	0.3437	-0.4601	-0.1058	0.2767	-0.1845	0.5406	-0.3342
**Lat**	-0.1000	1	-0.0988	-0.1468	-0.7249	0.2386	0.0754	-0.0330	0.0909	-0.0920	-0.1206
**PA_Prop**	0.1125	-0.0988	1	0.0404	0.1487	-0.1058	0.0180	0.0646	-0.2261	-0.0090	-0.0657
**Tmax_Ch**	-0.2086	-0.1468	0.0404	1	-0.0751	-0.0446	0.0345	-0.0467	-0.1351	-0.0561	0.1011
**Tmin_Ch**	0.3437	-0.7249	0.1487	-0.0751	1	-0.1838	-0.0670	0.0271	-0.0745	0.2718	-0.0076
**Precip_Ch**	-0.4601	0.2386	-0.1058	-0.0446	-0.1838	1	0.6513	-0.0463	-0.0012	-0.2378	0.0774
**Wetfreq_Ch**	-0.1058	0.0754	0.0180	0.0345	-0.0670	0.6513	1	0.2320	-0.2096	-0.1242	0.0486
**Access**	0.2767	-0.0330	0.0646	-0.0467	0.0271	-0.0463	0.2320	1	-0.2807	0.1586	-0.1416
**PopDens**	-0.1845	0.0909	-0.2261	-0.1351	-0.0745	-0.0012	-0.2096	-0.2807	1	-0.1033	0.0883
**GDP_Ch**	0.5406	-0.0920	-0.0090	-0.0561	0.2718	-0.2378	-0.1242	0.1586	-0.1033	1	-0.1357
**Pop_Ch**	-0.3342	-0.1206	-0.0657	0.1011	-0.0076	0.0774	0.0486	-0.1416	0.0883	-0.1357	1

Table B. Correlation matrix (Pearson correlation coefficient r) of model covariates on LAU level (*n* = 127102)

	**Long**	**Lat**	**PA_Prop**	**Tmax_Ch**	**Tmin_Ch**	**Precip_Ch**	**Wetfreq_Ch**	**Access**	**PopDens**	**Pop_Ch**
**Long**	1	-0.1584	0.0611	-0.0333	0.3771	-0.4920	-0.3384	0.2850	-0.1780	-0.0605
**Lat**	-0.1584	1	-0.0495	-0.1715	-0.6917	0.0972	-0.1387	-0.1085	0.1799	0.0559
**PA_Prop**	0.0611	-0.0495	1	0.0253	0.0337	-0.0712	-0.0165	0.1272	-0.0223	0.0180
**Tmax_Ch**	-0.0333	-0.1715	0.0253	1	-0.1257	-0.3387	-0.0508	-0.0332	-0.0100	0.0260
**Tmin_Ch**	0.3771	-0.6917	0.0337	-0.1257	1	0.0213	0.0995	0.1223	-0.1460	-0.0377
**Precip_Ch**	-0.4920	0.0972	-0.0712	-0.3387	0.0213	1	0.6839	-0.0410	0.0730	0.0452
**Wetfreq_Ch**	-0.3384	-0.1387	-0.0165	-0.0508	0.0995	0.6839	1	0.1703	-0.0551	0.0174
**Access**	0.2850	-0.1085	0.1272	-0.0332	0.1223	-0.0410	0.1703	1	-0.1570	-0.0376
**PopDens**	-0.1780	0.1799	-0.0223	-0.0100	-0.1460	0.0730	-0.0551	-0.1570	1	0.1671
**Pop_Ch**	-0.0605	0.0559	0.0180	0.0260	-0.0377	0.0452	0.0174	-0.0376	0.1671	1
