# Supplementary material for: Climatic and socioeconomic effects on land cover changes across Europe: Does protected area designation matter?
Source: PLoS One. 2019 Jul 17;14(7):e0219374. doi: 10.1371/journal.pone.0219374 (PMC6636817; doi:10.1371/journal.pone.0219374)
Supplement: S5 Appendix — (PDF) [file pone.0219374.s005.pdf]

S5 Appendix: Spatial distribution of land cover flows from 2000 to 2012

Table A. Areas of land cover flows (LCF1-LCF6) from 2000 to 2012 (km²) per biogeographical region [38], country and area type

Country	LCF1	LCF2	LCF3	LCF4	LCF5	LCF6	Total area LCF1-LCF6
Alpine	343.30	129.30	36.91	2135.01	3673.16	21.03	6338.71
Anatolian	605.49	440.30	75.40	211.96	108.40	297.56	1739.11
Arctic	64.88	18.38	0.00	71.58	17.63	251.03	423.50
Atlantic	2909.01	722.55	464.75	4981.62	7788.88	58.60	16925.41
Black Sea	144.58	23.26	10.41	300.48	216.76	45.65	741.14
Boreal	705.23	1060.67	393.30	30056.32	33713.40	4.75	65933.67
Continental	3216.25	1493.15	2201.21	4696.26	5569.08	176.24	17352.19
Mediterranean	4679.72	4299.69	1417.55	5923.28	10975.02	656.21	27951.47
Pannonian	367.28	747.31	730.63	2025.82	1626.39	34.83	5532.26
Steppic	20.80	7.05	11.18	3.44	31.33	9.19	82.99
Albania	292.91	11.17	8.07	86.89	309.61	15.35	724.00
Austria	138.74	0.28	1.03	25.23	475.85	1.02	642.15
Belgium	71.05	1.58	0.34	60.37	163.04	3.55	299.93
Bosnia and Herzegovina	101.14	55.23	4.57	182.42	235.57	3.27	582.20
Bulgaria	87.18	138.78	69.73	180.18	510.14	2.50	988.51
Croatia	140.04	130.40	5.48	135.82	486.49	2.98	901.21
Cyprus	130.99	30.36	14.60	112.03	30.58	0.40	318.96
Czech Republic	246.80	220.73	1563.87	709.89	434.21	4.13	3179.63
Denmark	192.59	22.80	15.15	112.35	128.67	14.24	485.80
Estonia	91.96	368.52	283.28	829.78	982.61	0.53	2556.68
Finland	217.58	178.32	0.00	5185.28	7231.67	0.00	12812.85
France	1654.63	270.75	55.23	2135.61	4600.37	19.43	8736.02
Germany	1055.61	883.11	370.72	582.46	749.61	102.90	3744.41
Greece	343.33	44.24	65.94	347.98	1029.81	30.66	1861.96
Hungary	245.53	590.16	619.68	1880.80	1388.66	19.58	4744.41
Iceland	65.68	18.69	0.00	72.29	17.69	273.78	448.13
Ireland	218.13	201.11	358.02	1229.39	692.51	0.00	2699.16
Italy	814.94	230.80	143.12	560.37	946.05	44.02	2739.30
Kosovo	36.68	27.03	10.15	47.96	31.91	0.00	153.73
Latvia	38.13	295.39	42.70	181.33	3112.27	1.01	3670.83
Liechtenstein	0.16	0.00	0.00	0.00	0.00	0.00	0.16
Lithuania	68.19	207.41	49.03	520.78	728.25	2.83	1576.49
Luxembourg	8.95	2.59	1.21	21.41	3.80	0.00	37.96
Macedonia	32.96	23.02	38.50	124.78	295.21	16.76	531.23
Malta	0.26	0.00	0.00	0.00	0.00	0.00	0.26
Montenegro	11.73	3.47	0.59	13.60	53.47	0.00	82.86
Netherlands	568.76	25.96	9.90	168.78	35.94	24.39	833.73
Norway	197.58	1.23	0.00	517.45	2899.68	0.00	3615.94
Poland	700.12	180.33	118.61	1800.39	1548.63	32.36	4380.44
Portugal	372.37	734.97	84.90	2532.98	4779.39	169.98	8674.59
Romania	204.02	91.51	43.03	74.81	937.15	10.15	1360.67
Serbia	75.70	90.81	59.49	172.13	195.77	21.52	615.42
Slovakia	100.11	29.09	74.63	329.19	1022.39	0.81	1556.22
Slovenia	15.70	0.09	0.06	4.12	21.59	0.53	42.09
Spain	2317.20	2949.15	958.22	1929.74	2951.78	216.11	11322.20
Sweden	290.69	12.36	25.00	24706.46	20219.93	0.76	45255.20
Switzerland	16.92	0.18	0.18	11.85	4.38	0.00	33.51
Turkey	1458.95	847.71	237.82	1705.71	2267.37	538.84	7056.40
United Kingdom	450.92	17.83	3.79	1107.21	2196.16	3.17	3779.08
Protected areas	789.65	1155.84	717.74	5953.23	9837.36	324.25	18778.07
Non-protected areas	12285.30	7781.33	4618.90	44446.63	53880.88	1253.31	124266.35
1 km protected area buffers	2783.85	1510.90	1009.63	10758.83	11054.13	157.51	27274.85
Total	13074.93	8937.16	5336.64	50399.82	63718.21	1577.56	143044.32

Table B. Areas of LCF1 (Urbanisation) from 2000 to 2012 (km²) per area type and biogeographical region [38] / country

Biogeographical region / Country	Protected areas	Non-protected areas	1 km protected area buffers	Total area
Alpine	35.71	307.59	62.59	343.30
Anatolian	0.00	605.49	0.21	605.49
Arctic	0.26	64.62	5.66	64.88
Atlantic	170.40	2738.61	727.18	2909.01
Black Sea	7.23	137.35	6.37	144.58
Boreal	8.65	696.58	186.03	705.23
Continental	260.89	2955.36	973.74	3216.25
Mediterranean	292.75	4386.97	717.94	4679.72
Pannonian	13.16	354.12	95.55	367.28
Steppic	1.50	19.30	4.85	20.80
Albania	19.83	273.08	11.44	292.91
Austria	13.81	124.93	41.54	138.74
Belgium	12.37	58.68	26.92	71.05
Bosnia and Herzegovina	0.00	101.14	1.59	101.14
Bulgaria	19.73	67.45	15.28	87.18
Croatia	37.85	102.19	31.46	140.04
Cyprus	18.36	112.63	16.18	130.99
Czech Republic	9.75	237.05	58.80	246.80
Denmark	7.60	184.99	184.03	192.59
Estonia	1.02	90.94	35.19	91.96
Finland	1.58	216.00	55.47	217.58
France	157.03	1497.60	334.20	1654.63
Germany	101.18	954.43	549.54	1055.61
Greece	60.85	282.48	59.77	343.33
Hungary	9.37	236.16	70.18	245.53
Iceland	0.26	65.42	5.73	65.68
Ireland	2.18	215.95	57.32	218.13
Italy	37.03	777.91	128.36	814.94
Kosovo	0.18	36.50	2.02	36.68
Latvia	1.30	36.83	4.90	38.13
Liechtenstein	0.00	0.16	0.02	0.16
Lithuania	3.54	64.65	18.29	68.19
Luxembourg	1.25	7.70	5.36	8.95
Macedonia	0.10	32.86	1.49	32.96
Malta	0.00	0.26	0.26	0.26
Montenegro	0.00	11.73	0.05	11.73
Netherlands	9.08	559.68	82.36	568.76
Norway	0.33	197.25	22.60	197.58
Poland	101.36	598.76	162.09	700.12
Portugal	32.46	339.91	39.62	372.37
Romania	6.05	197.97	28.74	204.02
Serbia	0.28	75.42	4.59	75.70
Slovakia	7.13	92.98	30.92	100.11
Slovenia	3.99	11.71	7.87	15.70
Spain	90.86	2226.34	474.74	2317.20
Sweden	1.77	288.92	84.76	290.69
Switzerland	0.29	16.63	6.83	16.92
Turkey	0.32	1458.63	0.54	1458.95
United Kingdom	19.54	431.38	122.80	450.92
Total	789.65	12285.30	2783.85	13074.93

Table C. Areas of LCF2 (Intensification of agriculture) from 2000 to 2012 (km²) per area type and biogeographical region [38] / country

Biogeographical region / Country	Protected areas	Non-protected areas	1 km protected area buffers	Total area
Alpine	39.98	89.32	17.55	129.30
Anatolian	0.00	440.30	0.12	440.30
Arctic	0.00	18.38	0.00	18.38
Atlantic	80.41	642.14	204.63	722.55
Black Sea	5.12	18.14	2.42	23.26
Boreal	31.58	1029.09	231.84	1060.67
Continental	348.98	1144.17	339.61	1493.15
Mediterranean	556.02	3743.67	519.71	4299.69
Pannonian	93.87	653.44	195.89	747.31
Steppic	0.28	6.77	1.34	7.05
Albania	0.23	10.94	1.44	11.17
Austria	0.00	0.28	0.11	0.28
Belgium	0.10	1.48	0.47	1.58
Bosnia and Herzegovina	0.00	55.23	0.24	55.23
Bulgaria	31.65	107.13	36.96	138.78
Croatia	55.73	74.67	23.61	130.40
Cyprus	15.31	15.05	4.20	30.36
Czech Republic	38.53	182.20	51.76	220.73
Denmark	5.74	17.06	17.06	22.80
Estonia	6.71	361.81	148.76	368.52
Finland	2.33	175.99	18.43	178.32
France	44.26	226.49	38.64	270.75
Germany	248.36	634.75	296.43	883.11
Greece	17.28	26.96	8.50	44.24
Hungary	79.48	510.68	167.63	590.16
Iceland	0.18	18.51	0.02	18.69
Ireland	2.45	198.66	43.76	201.11
Italy	22.71	208.09	31.27	230.80
Kosovo	0.00	27.03	0.63	27.03
Latvia	8.84	286.55	24.92	295.39
Liechtenstein	0.00	0.00	0.00	0.00
Lithuania	12.38	195.03	37.55	207.41
Luxembourg	0.77	1.82	1.45	2.59
Macedonia	0.00	23.02	0.31	23.02
Malta	0.00	0.00	0.00	0.00
Montenegro	0.00	3.47	0.06	3.47
Netherlands	0.29	25.67	3.61	25.96
Norway	0.00	1.23	0.00	1.23
Poland	43.58	136.75	29.43	180.33
Portugal	144.95	590.02	41.02	734.97
Romania	15.18	76.33	12.74	91.51
Serbia	2.30	88.51	8.29	90.81
Slovakia	3.75	25.34	7.84	29.09
Slovenia	0.00	0.09	0.09	0.09
Spain	344.50	2604.65	444.65	2949.15
Sweden	2.83	9.53	3.54	12.36
Switzerland	0.00	0.18	0.00	0.18
Turkey	1.07	846.64	1.30	847.71
United Kingdom	4.34	13.49	4.18	17.83
Total	1155.84	7781.33	1510.90	8937.16

Table D. Areas of LCF3 (Extensification of agriculture) from 2000 to 2012 (km²) per area type and biogeographical region [38] / country

Biogeographical region / Country	Protected areas	Non-protected areas	1 km protected area buffers	Total area
Alpine	10.26	26.65	5.24	36.91
Anatolian	0.00	75.40	1.31	75.40
Arctic	0.00	0.00	0.00	0.00
Atlantic	31.39	433.36	98.64	464.75
Black Sea	3.41	7.00	3.52	10.41
Boreal	26.57	366.73	131.57	393.30
Continental	419.98	1781.23	443.56	2201.21
Mediterranean	87.15	1330.40	150.11	1417.55
Pannonian	139.15	591.48	176.61	730.63
Steppic	0.00	11.18	1.35	11.18
Albania	2.12	5.95	0.16	8.07
Austria	0.22	0.81	0.44	1.03
Belgium	0.01	0.33	0.00	0.34
Bosnia and Herzegovina	0.00	4.57	0.36	4.57
Bulgaria	8.71	61.02	22.59	69.73
Croatia	0.28	5.20	0.42	5.48
Cyprus	3.26	11.34	1.00	14.60
Czech Republic	248.84	1315.03	285.13	1563.87
Denmark	3.05	12.10	12.10	15.15
Estonia	9.94	273.34	113.67	283.28
Finland	0.00	0.00	0.00	0.00
France	6.64	48.59	12.82	55.23
Germany	130.96	239.76	125.56	370.72
Greece	6.12	59.82	16.64	65.94
Hungary	125.41	494.27	152.11	619.68
Iceland	0.00	0.00	0.00	0.00
Ireland	7.03	350.99	64.83	358.02
Italy	4.77	138.35	8.46	143.12
Kosovo	0.00	10.15	0.09	10.15
Latvia	7.11	35.59	3.72	42.70
Liechtenstein	0.00	0.00	0.00	0.00
Lithuania	7.51	41.52	8.93	49.03
Luxembourg	0.00	1.21	0.84	1.21
Macedonia	0.00	38.50	0.26	38.50
Malta	0.00	0.00	0.00	0.00
Montenegro	0.00	0.59	0.00	0.59
Netherlands	0.46	9.44	0.42	9.90
Norway	0.00	0.00	0.00	0.00
Poland	47.79	70.82	19.45	118.61
Portugal	7.93	76.97	4.80	84.90
Romania	2.12	40.91	4.50	43.03
Serbia	1.40	58.09	6.56	59.49
Slovakia	18.07	56.56	14.56	74.63
Slovenia	0.00	0.06	0.00	0.06
Spain	63.17	895.05	119.85	958.22
Sweden	3.86	21.14	7.06	25.00
Switzerland	0.00	0.18	0.01	0.18
Turkey	0.00	237.82	1.42	237.82
United Kingdom	0.96	2.83	0.87	3.79
Total	717.74	4618.90	1009.63	5336.64

Table E. Areas of LCF4 (Afforestation) from 2000 to 2012 (km²) per area type and biogeographical region [38] / country

Biogeographical region / Country	Protected areas	Non-protected areas	1 km protected area buffers	Total area
Alpine	419.84	1715.17	356.16	2135.01
Anatolian	1.07	210.89	0.59	211.96
Arctic	0.59	70.99	3.41	71.58
Atlantic	1065.52	3916.10	1028.42	4981.62
Black Sea	7.19	293.29	1.13	300.48
Boreal	737.59	29318.73	7564.48	30056.32
Continental	2027.54	2668.72	813.43	4696.26
Mediterranean	1327.55	4595.73	535.99	5923.28
Pannonian	369.39	1656.43	458.98	2025.82
Steppic	2.48	0.96	0.00	3.44
Albania	20.96	65.93	11.64	86.89
Austria	4.94	20.29	7.47	25.23
Belgium	32.73	27.64	19.36	60.37
Bosnia and Herzegovina	0.06	182.36	2.47	182.42
Bulgaria	78.82	101.36	17.93	180.18
Croatia	74.20	61.62	13.46	135.82
Cyprus	47.78	64.25	22.45	112.03
Czech Republic	302.01	407.88	97.61	709.89
Denmark	15.95	96.40	96.34	112.35
Estonia	100.90	728.88	406.93	829.78
Finland	159.42	5025.86	950.17	5185.28
France	729.96	1405.65	388.84	2135.61
Germany	279.70	302.76	158.65	582.46
Greece	165.51	182.47	48.50	347.98
Hungary	315.55	1565.25	433.48	1880.80
Iceland	0.59	71.70	3.41	72.29
Ireland	210.49	1018.90	290.77	1229.39
Italy	193.24	367.13	103.24	560.37
Kosovo	3.27	44.69	1.75	47.96
Latvia	18.28	163.05	18.88	181.33
Liechtenstein	0.00	0.00	0.00	0.00
Lithuania	131.48	389.30	93.94	520.78
Luxembourg	14.02	7.39	5.64	21.41
Macedonia	14.78	110.00	5.69	124.78
Malta	0.00	0.00	0.00	0.00
Montenegro	0.00	13.60	0.00	13.60
Netherlands	40.86	127.92	26.85	168.78
Norway	6.20	511.25	53.63	517.45
Poland	1040.04	760.35	255.10	1800.39
Portugal	504.64	2028.34	164.21	2532.98
Romania	22.40	52.41	7.74	74.81
Serbia	42.13	130.00	10.87	172.13
Slovakia	187.34	141.85	45.70	329.19
Slovenia	2.92	1.20	1.03	4.12
Spain	516.75	1412.99	337.99	1929.74
Sweden	471.21	24235.25	6426.91	24706.46
Switzerland	1.66	10.19	6.75	11.85
Turkey	7.45	1698.26	6.57	1705.71
United Kingdom	194.98	912.23	216.85	1107.21
Total	5953.23	44446.63	10758.83	50399.82

Table F. Areas of LCF5 (Deforestation) from 2000 to 2012 (km²) per area type and biogeographical region [38] / country

Biogeographical region / Country	Protected areas	Non-protected areas	1 km protected area buffers	Total area
Alpine	1349.86	2323.30	413.41	3673.16
Anatolian	0.00	108.40	0.00	108.40
Arctic	0.34	17.29	0.24	17.63
Atlantic	2021.06	5767.82	1666.35	7788.88
Black Sea	15.28	201.48	3.87	216.76
Boreal	924.59	32788.81	6854.86	33713.40
Continental	2483.84	3085.24	834.76	5569.08
Mediterranean	2439.00	8536.02	994.42	10975.02
Pannonian	581.97	1044.42	288.39	1626.39
Steppic	24.14	7.19	1.28	31.33
Albania	35.62	273.99	21.04	309.61
Austria	132.82	343.03	93.59	475.85
Belgium	89.55	73.49	54.24	163.04
Bosnia and Herzegovina	0.08	235.49	5.82	235.57
Bulgaria	201.03	309.11	72.14	510.14
Croatia	239.54	246.95	64.70	486.49
Cyprus	25.69	4.89	3.99	30.58
Czech Republic	192.34	241.87	48.30	434.21
Denmark	33.51	95.16	94.56	128.67
Estonia	21.87	960.74	478.08	982.61
Finland	178.17	7053.50	1199.98	7231.67
France	1735.86	2864.51	862.30	4600.37
Germany	489.35	260.26	128.58	749.61
Greece	334.61	695.20	145.14	1029.81
Hungary	505.16	883.50	245.38	1388.66
Iceland	0.34	17.35	0.24	17.69
Ireland	122.17	570.34	221.79	692.51
Italy	309.06	636.99	159.27	946.05
Kosovo	0.84	31.07	0.19	31.91
Latvia	358.32	2753.95	335.34	3112.27
Liechtenstein	0.00	0.00	0.00	0.00
Lithuania	154.83	573.42	104.58	728.25
Luxembourg	2.68	1.12	0.75	3.80
Macedonia	7.74	287.47	11.43	295.21
Malta	0.00	0.00	0.00	0.00
Montenegro	0.04	53.43	0.05	53.47
Netherlands	17.76	18.18	1.66	35.94
Norway	14.02	2885.66	250.45	2899.68
Poland	921.82	626.81	182.01	1548.63
Portugal	818.99	3960.40	322.54	4779.39
Romania	432.23	504.92	94.88	937.15
Serbia	48.61	147.16	17.32	195.77
Slovakia	713.22	309.17	92.26	1022.39
Slovenia	16.96	4.63	2.96	21.59
Spain	960.43	1991.35	507.51	2951.78
Sweden	239.95	19979.98	4722.81	20219.93
Switzerland	0.11	4.27	2.09	4.38
Turkey	0.01	2267.36	3.78	2267.37
United Kingdom	482.03	1714.13	502.36	2196.16
Total	9837.36	53880.88	11054.13	63718.21

Table G. Areas of LCF6 (Formation of water bodies) from 2000 to 2012 (km²) per area type and biogeographical region [38] / country

Biogeographical region / Country	Protected areas	Non-protected areas	1 km protected area buffers	Total area
Alpine	2.26	18.77	2.05	21.03
Anatolian	0.00	297.56	0.00	297.56
Arctic	66.67	184.36	29.60	251.03
Atlantic	17.44	41.16	12.94	58.60
Black Sea	0.00	45.65	0.36	45.65
Boreal	0.88	3.87	0.71	4.75
Continental	79.35	96.89	36.43	176.24
Mediterranean	141.60	514.61	70.26	656.21
Pannonian	6.86	27.97	4.76	34.83
Steppic	9.18	0.01	0.01	9.19
Albania	5.24	10.11	0.70	15.35
Austria	0.60	0.42	0.13	1.02
Belgium	0.47	3.08	2.30	3.55
Bosnia and Herzegovina	0.00	3.27	0.04	3.27
Bulgaria	1.08	1.42	0.78	2.50
Croatia	0.92	2.06	0.10	2.98
Cyprus	0.35	0.05	0.05	0.40
Czech Republic	0.49	3.64	0.50	4.13
Denmark	13.87	0.37	0.37	14.24
Estonia	0.25	0.28	0.05	0.53
Finland	0.00	0.00	0.00	0.00
France	5.52	13.91	5.38	19.43
Germany	47.89	55.01	29.04	102.90
Greece	16.30	14.36	6.98	30.66
Hungary	6.27	13.31	3.51	19.58
Iceland	66.67	207.11	29.99	273.78
Ireland	0.00	0.00	0.00	0.00
Italy	31.04	12.98	2.41	44.02
Kosovo	0.00	0.00	0.00	0.00
Latvia	0.06	0.95	0.00	1.01
Liechtenstein	0.00	0.00	0.00	0.00
Lithuania	0.57	2.26	0.47	2.83
Luxembourg	0.00	0.00	0.00	0.00
Macedonia	0.60	16.16	0.96	16.76
Malta	0.00	0.00	0.00	0.00
Montenegro	0.00	0.00	0.00	0.00
Netherlands	4.27	20.12	2.84	24.39
Norway	0.00	0.00	0.00	0.00
Poland	12.81	19.55	6.19	32.36
Portugal	22.74	147.24	14.87	169.98
Romania	10.02	0.13	0.09	10.15
Serbia	1.37	20.15	0.96	21.52
Slovakia	0.18	0.63	0.41	0.81
Slovenia	0.07	0.46	0.08	0.53
Spain	73.71	142.40	46.30	216.11
Sweden	0.08	0.68	0.19	0.76
Switzerland	0.00	0.00	0.00	0.00
Turkey	0.00	538.84	0.41	538.84
United Kingdom	0.81	2.36	1.41	3.17
Total	324.25	1253.31	157.51	1577.56
