# Supplementary figures and images for: Climatic and socioeconomic effects on land cover changes across Europe: Does protected area designation matter?
Source: PLoS One. 2019 Jul 17;14(7):e0219374. doi: 10.1371/journal.pone.0219374 (PMC6636817; doi:10.1371/journal.pone.0219374)

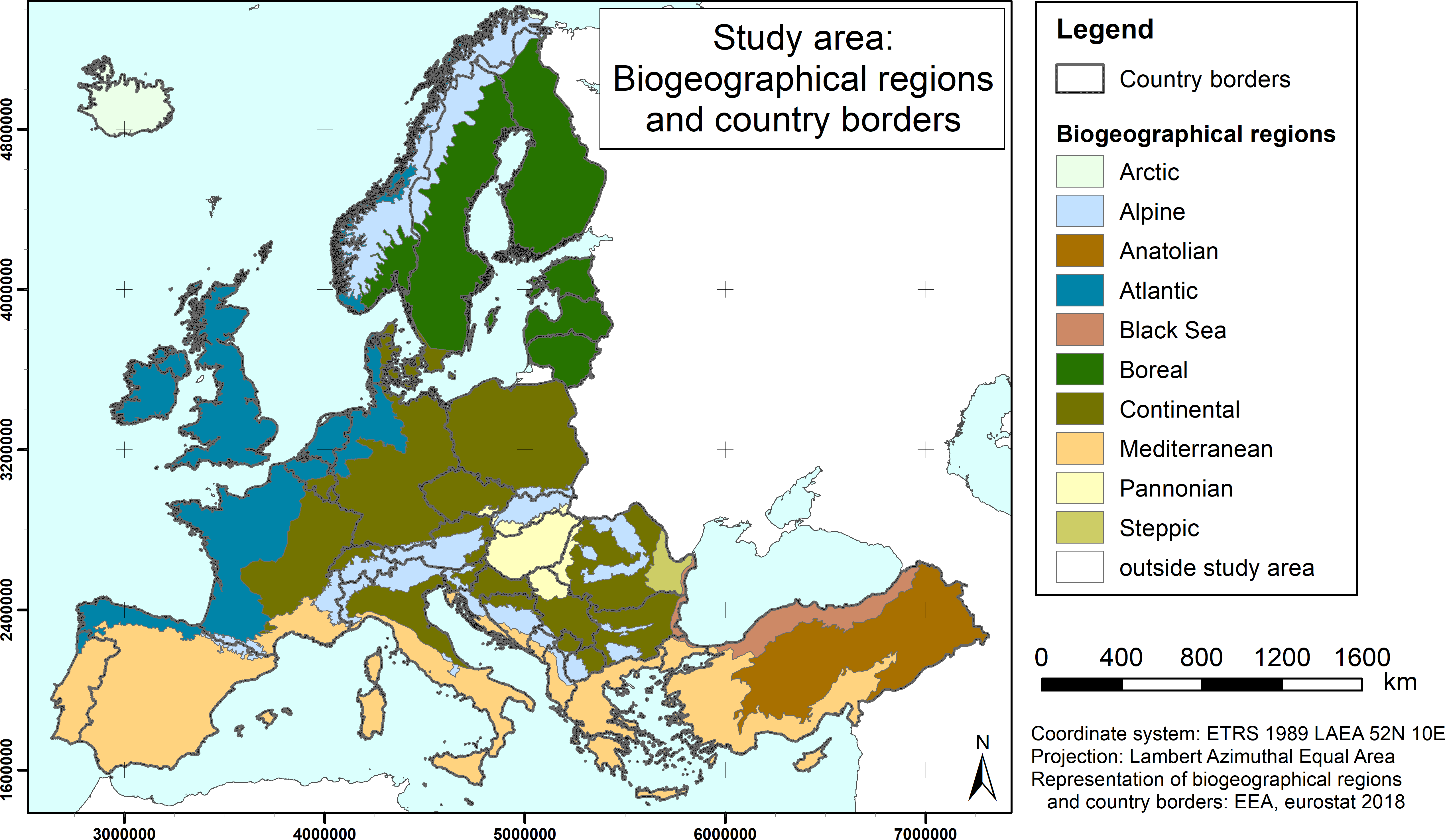

Supplement: S1 Fig — (TIF) [file pone.0219374.s006.tif]

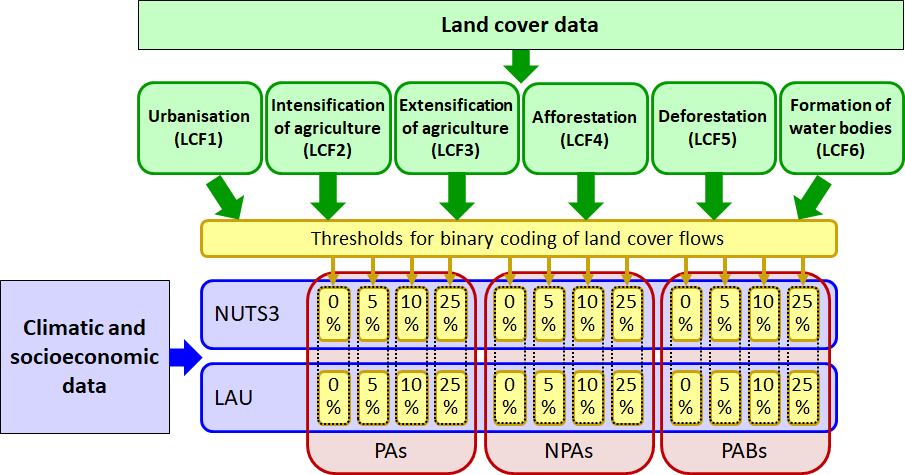

Supplement: S2 Fig — (TIF) [file pone.0219374.s007.tif]

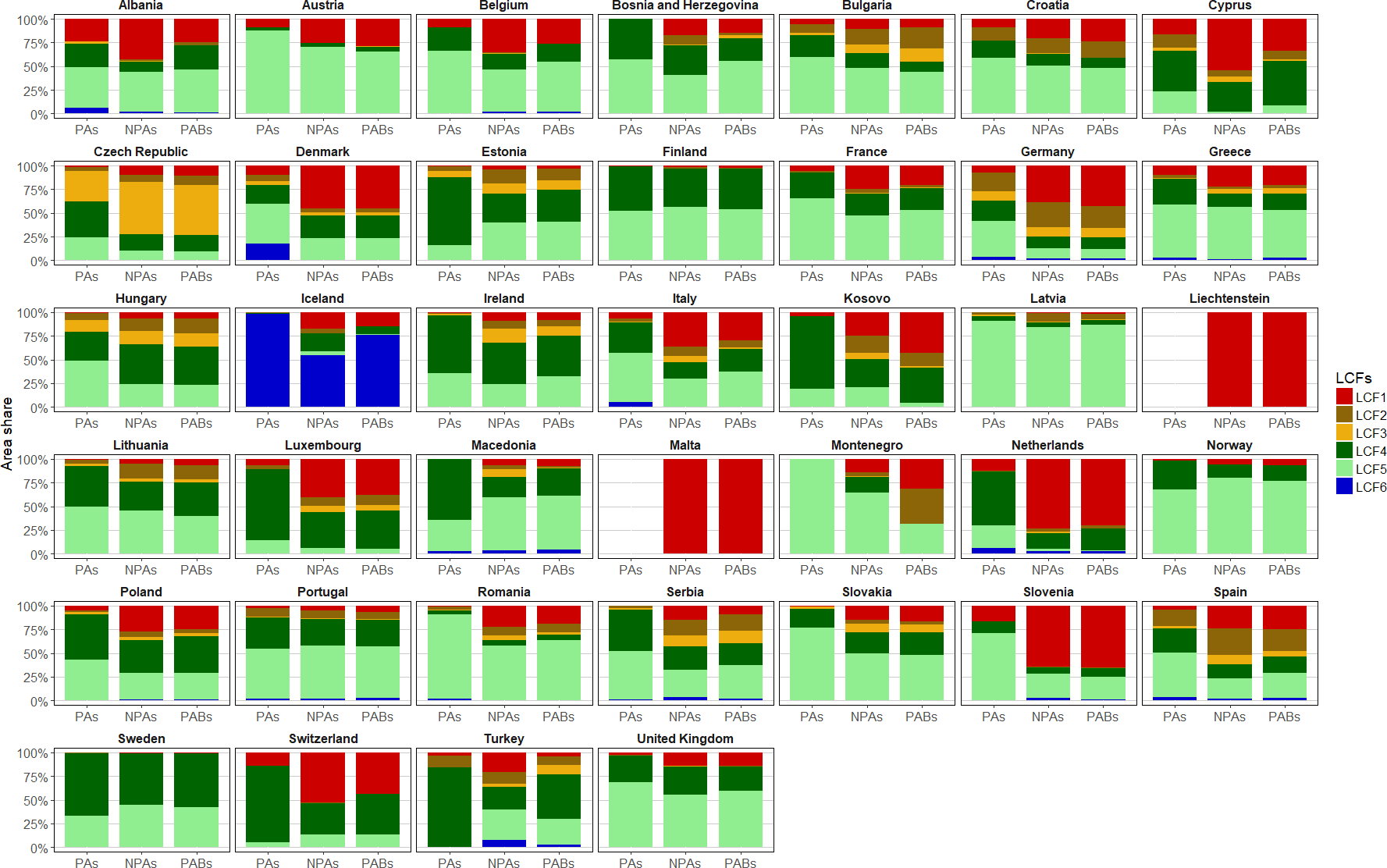

Supplement: S3 Fig — Area share values refer to the total areas of all land cover flows per area type and country. Total area values (in km2) are given in S5 Appendix. (TIF) [file pone.0219374.s008.tif]
